# Caecal microbial communities, functional diversity, and metabolic pathways in Ross 308 broiler chickens fed with diets containing different levels of Marama (*Tylosema esculentum*) bean meal

**DOI:** 10.3389/fmicb.2022.1009945

**Published:** 2022-10-20

**Authors:** Peter Kotsoana Montso, Caven Mguvane Mnisi, Ayansina Segun Ayangbenro

**Affiliations:** ^1^Food Security and Safety Focus Area, Faculty of Natural and Agricultural Sciences, North-West University, Mmabatho, South Africa; ^2^Department of Animal Science, School of Agricultural Sciences, Faculty of Natural and Agricultural Sciences, North-West University, Mmabatho, South Africa

**Keywords:** caecal microbiota, shotgun metagenomics sequencing, metabolic pathways, poultry, *Tylosema esculentum*

## Abstract

The caecum of a chicken harbors complex microbial communities that play vital roles in feed digestion, nutrient absorption, and bird health. Understanding the caecal microbial communities could help improve feed utilization efficiency and chicken product quality and, ultimately, deliver sustainable poultry production systems. Thus, this study assessed the caecal microbial communities and their functional diversity and metabolic pathways in broilers reared on diets containing different levels of marama (*Tylosema esculentum*) bean meal (MBM). A total of 350, day-old male Ross 308 broiler chicks were randomly allocated to five dietary treatments formulated as follows: a soybean-based standard broiler diet (Con_BC); Con_BC in which soybean products were substituted with 7 (M7_BC), 14 (M14_BC), 21 (M21_BC), and 28% (M28_BC) MBM. The dietary treatments were distributed to 35 replicate pens (10 birds each). After 42 days of feeding, the birds were slaughtered and thereafter caecal samples were collected from each replicate pen. Subsequently, the samples were pooled per treatment group for metagenomics sequence analysis. The results revealed that the bacteria domain (99.11%), with Bacteroides, Firmicutes and Proteobacteria being the most prominent phyla (48.28, 47.52, and 4.86%, respectively). Out of 846 genera obtained, the most abundant genera were *Bacteroides*, *Clostridium*, *Alistipes*, *Faecalibacterium*, *Ruminococcus*, *Eubacterium*, and *Parabacterioides.* At the genus level, the alpha-diversity showed significant (*p* < 0.05) difference across all treatment groups. Based on the SEED subsystem, 28 functional categories that include carbohydrates (14.65%), clustering-based subsystems (13.01%), protein metabolism (10.12%) were obtained. The KO analysis revealed 183 endogenous pathways, with 100 functional pathways associated with the metabolism category. Moreover, 15 pathways associated with carbohydrates were observed. The glycolysis/gluconeogenesis, galactose metabolism, pyruvate metabolism (15.32, 12.63, and 11.93%) were the most abundant pathways. Moreover, glycoside hydrolases (GH1, GH5, and GH13) were the most prominent carbohydrates-active enzymes. Therefore, results presented in this study suggest that dietary MB meal can improve microbial communities and their functional and metabolic pathways, which may help increase poultry production.

## Introduction

The global human population is projected to reach 9.6 billion in 2050 (Borda-Molina et al., 2018), which suggests that the demand for animal products *per capita* will inevitably increase. Indeed, the global consumption of poultry products (meat and eggs) is anticipated to increase by 73% by 2050 (McLeod, 2011), indicating that poultry products are the most consumed commodity worldwide. However, to meet the demand for the rapidly growing human population, poultry producers must employ the most efficient, sustainable, and cost-effective strategies to optimize production. Unfortunately, these efforts are usually hampered by high feed cost, which constitutes more than 70% of the total cost of production (Thirumalaisamy et al., 2016). The main factor contributing to high feeding costs is the over-reliance on maize and soybean to formulate poultry feeds (Selaledi et al., 2020). These two conventional ingredients have direct food value for humans and are also used for several applications in the food, feed, and biofuel sectors. As a result, their high demand has also resulted in a rise in their market prices, which poses severe financial consequences for the poultry industry. This suggests a need to consider the utilization of other non-conventional protein sources such as marama (*Tylosema esculentum*) bean meal (MBM). Marama bean plant is a drought-tolerant, naturally growing, and locally available legume that has an outstanding nutritional profile similar to soybean plant (Tibe et al., 2016).

Apart from its high crude protein (29–39%) content, marama beans contain bioactive compounds such as vitamins, phenolic acids, flavonoids and phytosterols that have antimicrobial peptides, antioxidant, and immunomodulatory properties (Amonsou et al., 2012). These bioactive substances have been reported to reduce pathogenic microbes (*Escherichia coli*, *Clostridium*, and *Salmonella* species) in the lower gastrointestinal tract (GIT) of a chicken (Swelum et al., 2021; Abd El-Hack et al., 2022). Similar studies have reported that the inclusion of phytogenic products in poultry diets can stimulate digestion, improve gut microbial activity, and increase immune response and gut health (Stastnik et al., 2021).

The lower GIT of a chicken harbors complex microbial communities that play a vital role in poultry nutrition, health, and growth performance (Zhao et al., 2013; Yadav and Jha, 2019). In addition, these gut microbes stimulate digestion, nutrient absorption, and strengthen the development of the immune system in poultry (Singh et al., 2012). The chicken GIT harbors over 900 bacteria species (Deusch et al., 2015; Borda-Molina et al., 2018). However, the microbial composition within the chicken GIT varies according to location (e.g., crop, gizzard, intestines, caeca, and colon), nutrition, genetic, age and other environmental factors, with nutrition being the most critical factor that influences the gut microbiota (Tilocca et al., 2016; Borda-Molina et al., 2018; Clavijo and Flórez, 2018; Yadav and Jha, 2019). The caeca harbor a large microbial population, with more than 900 bacteria species in 100 genera and 600, 000 genes (Broom and Kogut, 2018; Clavijo and Flórez, 2018; Marmion et al., 2021). In contrast with the upper GIT where feed passage takes only 2.5 h (Singh et al., 2012), feed stays for 12–20 h in the caecum allowing colonization by a diverse group of microorganisms (Borda-Molina et al., 2018; Broom and Kogut, 2018; Marmion et al., 2021). In addition, most caecal microbes are associated with high feed efficiency and production of short chain fatty acids, which signifies the importance of this organ in poultry (Yan et al., 2017; Borda-Molina et al., 2018). However, several gut microbes are non-culturable on laboratory media due to their unknown nutrient requirements (Shang et al., 2018), which has necessitated the need to employ high throughput sequencing method such as 16S rRNA-based next-generation sequence approach to explore the microbial population in various environments (Deusch et al., 2015).

The whole metagenomics shotgun (WMS) sequencing analysis is a powerful method that offers comprehensive and high-resolution microbial community data, especially from a complex environment such as the GIT (Stanley et al., 2013; Xu et al., 2021). Contrary to culture-dependent method, WMS analysis can identify microbial structure and their functional roles in the ecosystem (Thomas et al., 2017; Kumar et al., 2018; Chen et al., 2022; Yu et al., 2022). Furthermore, this method can identify gene sequences associated with enzymes responsible for the metabolism of carbohydrates, amino acids, minerals, and vitamins. Given that the caecum harbors the largest microbial population and retains feed for 12 to 20 h (Broom and Kogut, 2018), several studies have used this organ to investigate microbial structure functional diversity and metabolic pathways using shotgun metagenomics sequence analysis (Delgado et al., 2019; Qi et al., 2019). However, there are currently no studies that have investigated the microbial population and functional diversity of Ross 308 broiler chickens reared on diets containing different levels of MBM. Therefore, this study investigated the caecal microbial population and their functional diversity and metabolic pathways in broilers fed with MBM-containing diets using whole metagenomics approach. It was hypothesized that the inclusion of different levels on MBM in place of soybean products would improve the caecal microbial population and their functional diversity and metabolic pathways of the birds.

## Materials and methods

### Animals, experimental design, and dietary treatments

A total of 350 day-old male Ross 308 broiler chicks bought from Superior Chicks (PTY) Ltd. (Gauteng, South Africa) were evenly distributed to 35 replicate pens (3.5 m Length × 1.0 m Width × 1.85 m Height) in Rooigrond Farm (North West, South Africa). The chicks were raised using a commercial starter meal for the first 2 weeks and orally supplemented with a stress control (containing vitamins and electrolytes) for the first 3 days. The birds were then introduced to the experimental diets from day 14–42 during which growth measurements were taken. The dietary treatments were formulated using a standard broiler diet for the grower (14–28 days) and finisher (29–42 days) phases as follows: a soybean-based standard broiler diet (Con_BC); Con_BC in which soybean products were substituted with 7 (M7_BC), 14 (M14_BC), 21 (M21_BC), and 28% (M28_BC) of MBM. Each dietary treatment had 7 replicates with 10 birds per pen, which were designated as the experimental unit. The floor pens were covered with sunflower husk as bedding. All the pens were fitted with a feeder and water drinker that were regularly cleaned and filled with the dietary treatments and clean water, respectively. The dietary treatments and water were supplied *ad libitum* until day 42. At day 42 of age, the chickens were taken to a local abattoir where they were rendered unconscious by electrically stunning them and then slaughtered by cutting the jugular vein with a knife.

### Sample collection

After slaughter, the carcasses were manually eviscerated and caecal samples were collected from each carcass per pen. The collected samples were immediately pooled per dietary treatment to obtain 5 composite samples that had equal portions (approximately 5 g) of 5 individual fresh caecal contents. The samples were placed into 50 ml sterile falcon tubes and were snap frozen using liquid nitrogen and stored at −80°C for further analysis.

### Metagenomics DNA extraction, library preparation, and sequencing

The samples were thawed at room temperature for 30 min before the DNA extraction process was performed. Total metagenomics DNA was extracted from all composite samples using the QIAamp DNeasy PowerFecal DNA Kit (Qiagen, Germantown, MD) according to the manufacturer’s instructions. The DNA quality was determined using NanoDrop spectrophotometer (Thermo Fisher Scientific). The DNA sample with a ratio between 1.8 and 2.0 (260/280) and a 2.0 and 2.2 (260/230) was considered pure and suitable for sequencing. The DNA concentration for all five samples ranged between 21.0 and 82.7 ng/μL. The shotgun metagenomics sequencing was performed at the CosmosID, Germantown, US. Briefly, DNA libraries (1 ng input) were prepared using Nextera XT DNA Library Preparation Kit (Illumina, Sa Diego CA, United States) and IDT Unique Dual Indexes, following the standard procedure as stated by the manufacturer. Genomic DNA was fragmented using Nextera XT fragmentation enzyme (Illumina). After adding unique dual indexes, samples underwent 12 cycles of PCR to construct libraries. Subsequently, DNA libraries were purified using AMpure magnetic beads (Beckman Coulter, Brea CA, United States) followed by elution in Qiagen EB buffer. Final DNA libraries were quantified using a Qubit 4 fluorometer and Qubit™ dsDNA HS Assay Kit. Libraries were then sequenced on an Illumina HiSeq 4,000 platform 2 × 150 bp and paired reads were generated.

### Bioinformatics analysis

The raw reads were uploaded into the metagenomics rapid annotations Using Subsystem Technology (MG-RAST) online server^1^ (v 4.0.3; Meyer et al., 2008). Subsequently, joined paired-end reads were subjected to quality control (QC) on MG-RAST portal. Briefly, the QC filtering process involves dereplication, which includes removal of artificial duplicates sequences resulting from sequence artifacts, sequence reads with more than two times standard deviation above the mean reads length, host specific-species sequences, and trimming of adapter and low-quality sequences (sequences with more than five ambiguous base pair and 15 pH red score cut-off score; Gomez-Alvarez et al., 2009; Cox et al., 2010). The cleaned raw sequences were subjected to BLAST-like alignment tool (BLAT) algorithm (Kent, 2002), against M5NR database, which permits non-redundant incorporation of several databases (Wilke et al., 2012). No further analysis was performed on the sequences that could not pass annotation. The data was further normalized to reduce the effect of experimental error/noise. Taxonomic classification was performed using the NCBI RefSeq database (which provides a comprehensive, integrated, non-redundant and well-annotated set of sequences). The data were further subjected to the SEED subsystem and Kyoto Encyclopedia of Genes and Genomes (KEEG) Orthology (KO) databases to identify the functional categories at level 1 and 3 as well as the metabolic pathways with all parameters set at default (e-value = 1 × 10^−5^, minimum alignment length = 15 bp and identity = 60%). The enzymes involved in carbohydrates, amino acids and vitamins metabolism were manually curated and linked to the microbes found in the samples. The carbohydrate-active enzymes (CAZymes) database was used to identify CAZymes at the family level with all parameters set at default. The raw reads were deposited into the NCBI database under Bioproject accession number PRJNA818149 for all caecal samples with Sequence Read Archive (SRA) accession numbers: SRX15299735, SRX15299736, SRX15299737, SRX15299738 and SRR19238953 (Con_BC, M7_BC, M14_BC, M21_BC and M28_BC, respectively).

### Statistical analysis

After analyzing the data using MG-RAST portal, the files were exported to Microsoft Excel to compute the abundance and distribution of the microbiota at the phyla and genera levels. The heatmaps for the relative abundance of microbiota, functional roles and metabolic pathways were drawn using ClustVis^2^ (Metsalu and Vilo, 2015), with all parameters set at default. The Kruskal-Wallis test, a nonparametric statistical test, was performed to compare the alpha diversity indices (Simpson, Shannon, and Evenness) of each group, while one-way analysis of similarities (ANOSIM) through 9,999 permutation was used to compute beta-diversity (P < 0.05) in PAST (v3.20; Hammer et al., 2001). The abundance of microbial communities (phylum and genus level) among the treatment groups was determined using the linear discriminant analysis (LDA) effect size (LEfSe) on galaxy platform (version1.0), with a threshold score set at 2.0^3^ (Segata et al., 2011).

## Results

### Metagenomics sequence analysis

**Figure 1:**
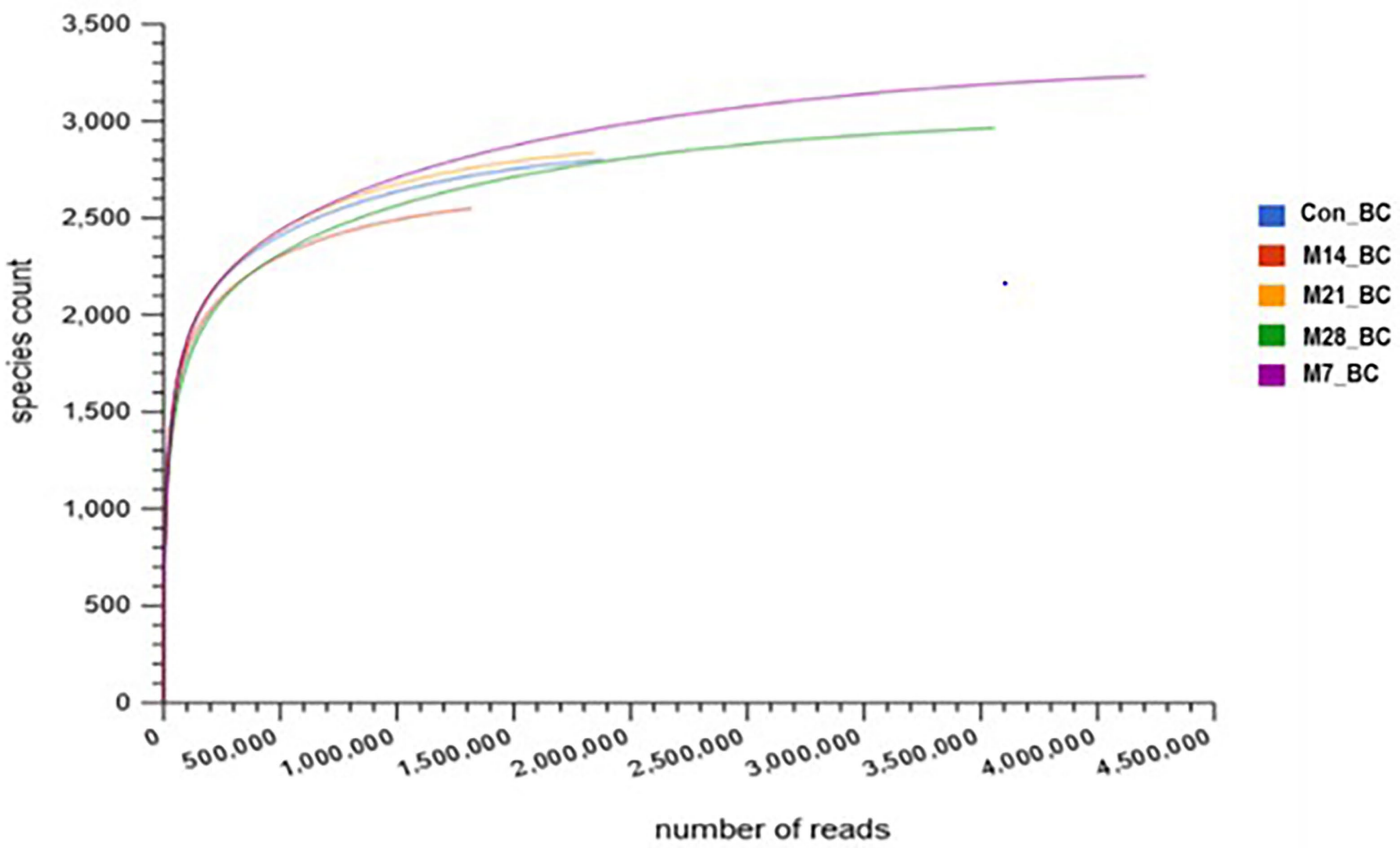
Rarefaction curve showing the microbial community richness in caecal digesta from broiler chicken fed with diets containing different levels of marama bean meal [Treatment groups: 0% (Con_BC); 7% (M7_BC); 14% (M14_BC); 21% (M21_BC); 28% (M28_BC) inclusion levels].

For the 28-day feeding period, each bird consumed a total of 3163.3–3330.8 g of feed and gained between 1556.0 and 2091.1 g of body mass. This translated to a feed conversion ratio of 1.60–2.08. The number of raw reads for each sample (Con_BC = 1,880,850, M7_BC = 4,205,050, M14_BC = 1,319,561, M21_BC = 1,843,022, and M28_BC = 3,557,783) uploaded into MG-RAST server are presented in Supplementary Table S1. After QC, a total of 8,680,093 sequences (Con_BC = 1,302,880, M7_BC = 2,790,421, M14_BC = 912,871, M21_BC = 1,379,900, and M28_BC = 2,294,021) were retained, Supplementary |Table S1. The average GC content ranged from 54.18 ± 9.995 to 55.874 ± 10.204. The RNA features were Con_BC = 11,920, M7_BC = 31,096, M14_BC = 9,254, M21_BC = 10,500, and M28_BC = 19,781, while protein coding features were Con_BC = 1,303,369, M7_BC = 2,783,978, M14_BC = 911,309, M21_BC = 1,377,138, and M28_BC = 2,284,533, Supplementary Table S1. As depicted in Figure 1, the rarefaction curves for all samples reached their plateau.

### Taxonomic composition of the microbial communities in the caecum digesta samples

**Figure 2:**
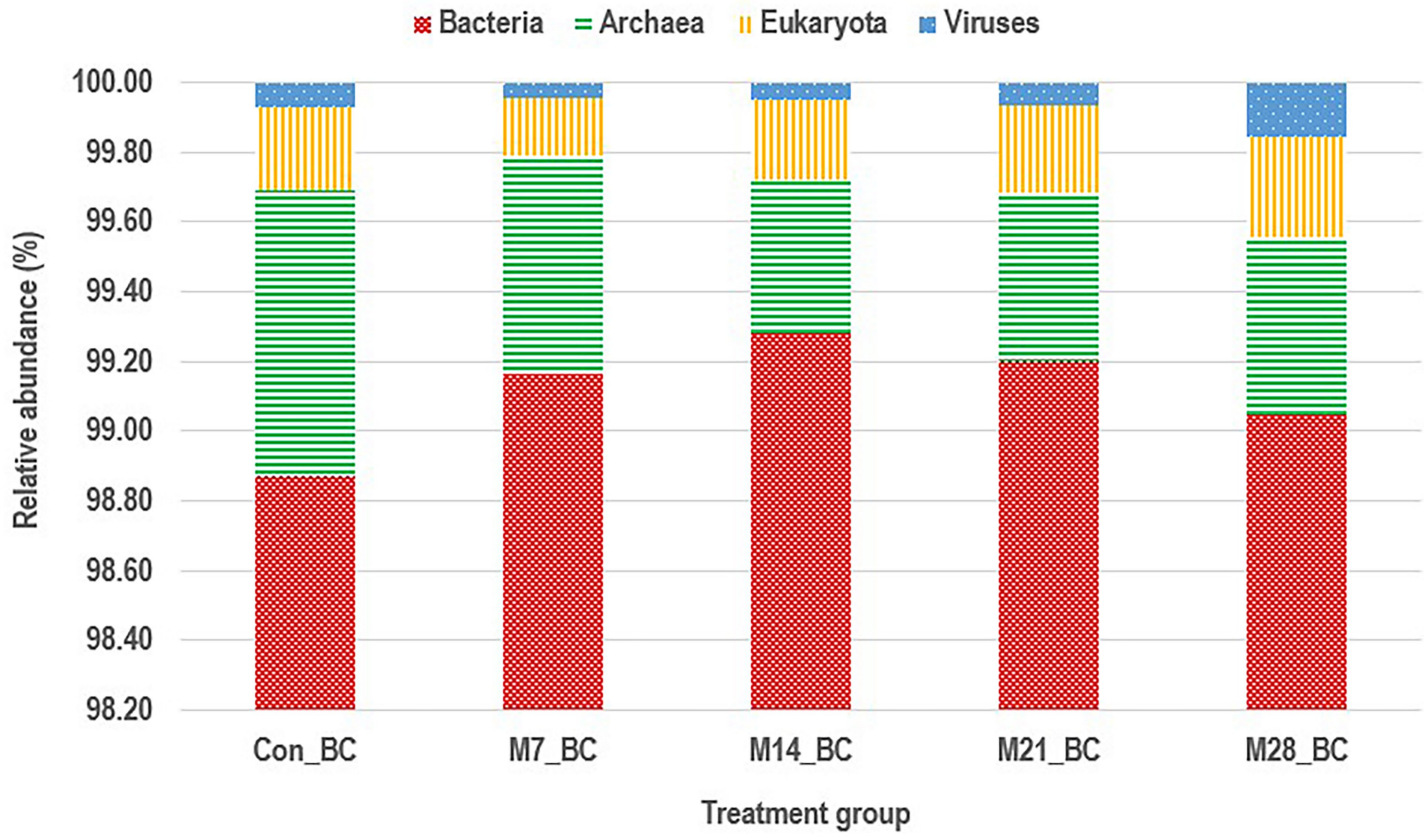
Stacked bar chart showing the major domain of microbial communities in caecum digesta samples from broiler chickens fed with diets containing different levels of marama bean meal [Treatment groups: 0% (Con_BC); 7% (M7_BC); 14% (M14_BC); 21% (M21_BC); 28% (M28_BC) inclusion levels].

Based on MG-RAST NCBI RefSeq database, a total of 8,215,033 sequences were classified into four major domains, Figure 2. A large proportion (99.11%) of the sequences were assigned to bacteria domain while the remaining sequences were ascribed to Archaea (0.58%), Eukaryota (0.23%) and Viruses (0.08%) domains. The treatment (MBM) groups revealed that high (99.05 to 99.28%) sequences linked to bacteria domain compared to the control (98.87%) group. However, there was no significant difference in microbial composition between the MBM and CON groups. Fifty-two phyla were identified and 16 of the most abundant (top 20) phyla belonged to bacteria domain. On average, the most predominant bacteria phyla were Firmicutes (48.28%), Bacteroides (47.52%) and Proteobacteria (4.89%) in the caecum digesta samples. As shown in Table 1, high (60.71%) microbial composition (Firmicutes) was observed in the M21_BC treatment group. Two Archaea phyla, Euryarchaeota (0.58%) and Crenarchaeota (0.02%), and two Eukaryota phyla Ascomycota (0.04%) and Basidiomycota (0.01%), were relatively predominant in all samples. At the class level, 120 classes were identified and a large proportion (85%) of the sequences were categorized under known classes, while 15% were grouped as ‘unclassified’. Bacteroidia, Clostridia and Bacilli were the most dominant classes (39.32, 38.12 and 4.15%, respectively). At the family level, 409 families were observed and 86.31% of the sequences were ascribed to known families, whereas 13.69% were grouped as “unclassified” families. On average, the most predominant families were Bacteroidaceae (24.22%), Ruminococcaceae (15.76%), Clostridiaceae (12.02%), Rikenellaceae (8.56%), and Porphyromonadaceae (3.68%) in all samples. At the genus level, 846 genera were obtained (Con_BC = 94.44%, M7_BC = 96.22%, M14_BC = 94.21%, M21_BC = 93.50% and M28_BC = 94.44%). The most abundant (average > 2%) genera among the top 20 were *Bacteroides*, *Clostridium*, *Alistipes*, *Faecalibacterium*, *Ruminococcus*, *Eubacterium*, and *Parabacterioides* (24.22, 11.56, 8.54, 7.62, 3.39, 2.84, and 2.10% respectively). As illustrated in Table 2, high abundance (28.77%) of the *Bacteroides* genus was observed in the M7_BC treatment groups.

**Table 1:** Relative abundance (%) of the dominant phyla (top 20) in caecum digesta samples from broiler chickens fed with diets containing different levels of marama bean meal.

Phylum	Con_BC	M7_BC	M14_BC	M21_BC	M28_BC
Firmicutes	49.48	48.25	51.77	60.71	31.08
Bacteroidetes	36.54	37.97	36.99	67.88	58.18
Proteobacteria	6.47	4.61	5.13	5.98	4.88
Actinobacteria	2.34	5.47	2.26	3.53	1.64
Euryarchaeota^*^	0.78	0.60	0.42	0.66	0.49
Verrucomicrobia	0.58	0.23	0.17	0.27	0.26
Spirochaetes	0.57	0.45	0.51	0.63	0.40
Fusobacteria	0.54	0.45	0.48	0.65	0.44
Synergistetes	0.43	0.32	0.17	0.22	0.13
Cyanobacteria	0.34	0.26	0.30	0.43	0.33
Chlorobi	0.28	0.13	0.21	0.40	0.46
Chloroflexi	0.27	0.24	0.24	0.40	0.24
Thermotogae	0.17	0.16	0.17	0.25	0.17
Deferribacteres	0.12	0.06	0.19	0.07	0.07
Deinococcus-thermus	0.12	0.09	0.10	0.16	0.13
Fibrobacteres	0.10	0.09	0.08	0.11	0.07
Acidobacteria	0.09	0.07	0.09	0.14	0.11
Ascomycota^**^	0.04	0.03	0.04	0.06	0.03
Crenarchaeota^*^	0.03	0.02	0.02	0.03	0.02
Basidiomycota^**^	0.01	0.01	0.01	0.01	0.01

The asterisks (^*^ and ^**^) denote phyla belonging to Archaea and Eukaryota domains, respectively. Treatment groups: 0% (Con_BC); 7% (M7_BC); 14% (M14_BC); 21% (M21_BC); 28% (M28_BC) inclusion levels.

**Table 2:** Relative abundance (%) of the dominant genera (top 20) in caecum digesta samples from broiler chickens fed with diets containing different levels of marama bean meal.

Genus	Con_BC	M7_BC	M14_BC	M21_BC	M28_BC
*Bacteroides*	22.71	28.77	21.32	24.85	23.48
*Clostridium*	12.47	12.99	12.45	11.72	8.17
*Faecalibacterium*	10.84	5.73	13.40	3.57	4.55
*Alistipes*	3.98	1.48	8.01	11.17	18.06
*Eubacterium*	2.88	3.47	3.02	2.95	1.87
*Ruminococcus*	2.85	4.18	2.97	3.89	3.06
*Parabacteroides*	2.39	1.58	1.73	1.93	2.86
*Desulfovibrio*	2.07	1.40	0.70	0.57	0.51
*Prevotella*	2.06	4.26	1.45	3.42	2.63
*Subdoligranulum*	1.73	1.69	1.95	2.58	1.02
*Ethanoligenens*	1.23	0.97	1.27	0.91	0.63
*Porphyromonas*	1.00	0.29	0.69	0.90	1.93
*Bacillus*	0.89	0.85	0.94	1.00	0.88
*Holdemania*	0.82	0.76	0.84	0.72	0.53
*Butyrivibrio*	0.71	0.76	0.70	0.68	0.39
*Desulfitobacterium*	0.65	0.67	0.58	0.57	0.38
*Anaerotruncus*	0.65	0.56	0.60	0.51	0.34
*Streptococcus*	0.64	1.34	0.69	0.72	0.51
*Roseburia*	0.63	0.70	0.76	0.56	0.36
*Blautia*	0.56	1.23	0.48	0.87	0.40

Treatment groups: 0% (Con_BC); 7% (M7_BC); 14% (M14_BC); 21% (M21_BC); 28% (M28_BC) inclusion levels.

### Alpha and beta diversity of microbial communities in the caecum digesta samples

The alpha diversity of microbial communities at phylum and genus levels was assessed using the Simpson, Shannon, and Evenness indices. At the phylum level, both Kruskal-Wallis rank-sum and LefSE analyses revealed no significant difference in the diversity and richness of the microbial population among the samples. However, at the genus level, Kruskal-Wallis rank-sum test (*p* < 0.05) showed there was a significant difference in the diversity and richness of genera detected across all samples (MBM and CON; (Table 3). However, the LefSE analysis revealed no significant difference (*P* ˃ 0.05). For the beta-diversity, the analysis of similarities (ANOSIM) revealed that there was no significant difference (*P* ˃ 0.05 and *R* = 0.011) in the microbial communities at both phylum and genus level between the treatment groups and control.

**Table 3:** The estimation of alpha diversity indices of microbial communities in caecum digesta samples from broiler chickens fed with diets containing different levels of marama bean meal.

Taxon	Indices	Con_BC	M7_BC	M14_BC	M21_BC	M28_BC	*P*-Value
Phylum	Simpson_1-D	0.62	0.62	0.59	0.60	0.56	2.673 × 10^−1^
	Shannon_H	1.28	1.25	1.19	1.18	1.16	
	Evenness_e^H/S	0.07	0.07	0.06	0.06	0.06	
Genus	Simpson_1-D	0.91	0.89	0.91	0.90	0.90	2.334 × 10^−20^
	Shannon_H	3.73	3.48	3.59	3.60	3.58	
	Evenness_e^H/S	0.05	0.04	0.05	0.05	0.05	

Treatment groups: 0% (Con_BC); 7% (M7_BC); 14% (M14_BC); 21% (M21_BC); 28% (M28_BC) inclusion levels.

### Microbial function analysis

**Figure 3:**
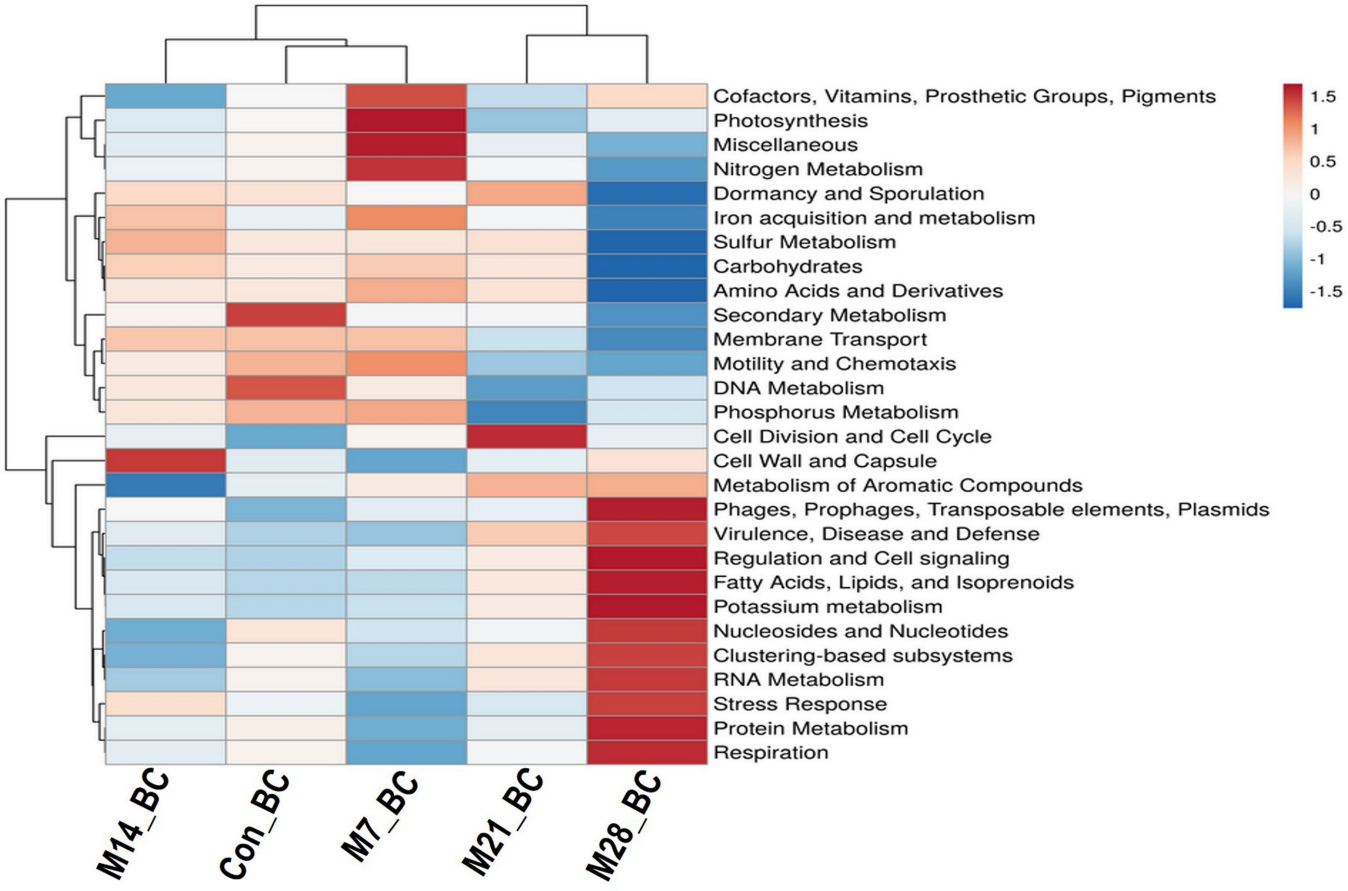
The heatmap showing relative abundance (mean > 0.02%) of the major functions (at level 1) of the microbial communities in caecum digesta samples from broiler chickens fed with diets containing different levels of marama bean meal The *X*-axis shows the treatment groups [Treatment groups: 0% (Con_BC); 7% (M7_BC); 14% (M14_BC); 21% (M21_BC); 28% (M28_BC) inclusion levels] while *Y*-axis represents metabolism pathways. The scale bar shows the colour saturation gradient dependent on the relative abundance. The colour intensity in each panel reflects relative abundances used for analysis (blue: low, white: medium, red: high).

The data were subjected to the SEED subsystem database to determine and uncover functional modules enriched in the caecal digesta samples. Based on the subsystem database (level 1), the sequences were assigned to 28 functional categories, as shown in Figure 3. Among these, functional categories associated with carbohydrates (14.65%), clustering-based subsystems (13.01%), protein metabolism (10.12%), amino acids and derivatives (8.22%), DNA metabolism (6.63%), miscellaneous (5.43%), RNA metabolism (4.94%), cofactors, vitamins, prosthetic groups, pigments (4.52%), cell wall and capsule (4.51%), nucleosides and nucleotides (4.04%), respiration (3.41%), and virulence, diseases, and defence (3.20%) predominated the caecum digesta samples. The abundance of other functional categories was below 3%. Subsystem level 3 revealed 1,010 functional categories (Con_BC = 92.38%, M7_BC = 95.64%, M14_BC = 85.84%, M21_BC = 88.81% and M28_BC = 87.92%), which were linked to 28 subsystem categories observed at Subsystem level 1. The categories DNA_repair_UvrABC_system (1.06%), Ribosome_LSU_bacterial (1.20%), Sugar_utilization_in_Thermotogales (1.1%) were the most predominant among the selected top 20 functional categories across all samples, Supplementary Figure S2; Supplementary Table S2.

### Alpha and beta diversity of functional categories in the caecum digesta samples

The alpha and beta diversity of the functional categories were assessed using the Simpson, Shannon and Evenness indices. The Kruskal-Wallis rank-sum test showed that there was a significant difference on the diversity and richness of functional categories observed at level 1 and 3 among the treatment groups (*p* < 0.05), as indicated in Table 4. However, β-diversity analysis of similarities (ANOSIM) indicated that there was no difference (*P* ˃ 0.05 and *R* = 0.011) in the richness of functional categories (level 1 and 3) between the treatment groups and control.

**Table 4:** The estimation of alpha diversity indices of functional categories of microbial community in caecum digesta samples from broiler chickens fed with diets containing different levels of marama bean meal.

Functional category	Indices	Con_BC	M7_BC	M14_BC	M21_BC	M28_BC	*P*-Value
Level 1	Simpson_1-D	0.92	0.92	0.92	0.92	0.93	2.524 × 10^−2^
	Shannon_H	2.81	2.82	2.82	2.82	2.83	
	Evenness_e^H/S	0.60	0.60	0.60	0.60	0.61	
Level 3	Simpson_1-D	1.00	1.00	1.00	1.00	1.00	1.642 × 10^−30^
	Shannon_H	5.97	5.97	5.93	5.94	5.86	
	Evenness_e^H/S	0.42	0.41	0.44	0.43	0.40	

Treatment groups: 0% (Con_BC); 7% (M7_BC); 14% (M14_BC); 21% (M21_BC); 28% (M28_BC) inclusion levels.

### Functional prediction of microbial communities in the caecum digesta samples

The data were subjected to the KO database to determine metabolic pathways enriched in the caecum digesta samples. The sequences were assigned to six major functional categories (KO level 1) related to metabolism (58.61%), genetic information processing (24.69%), environmental information processing (11.96%), cellular processes (3.20%), human diseases (1.07%), and organismal systems (0.47%). A total of 11 endogenous pathways (KO level 2) belonging to metabolism (KO functional category level 1) were associated with amino acid metabolism, carbohydrate metabolism, nucleotide metabolism, metabolism of cofactors and vitamins, energy metabolism, glycan biosynthesis and metabolism, lipid metabolism, biosynthesis of other secondary metabolites, metabolism of terpenoids and polyketides, metabolism of other amino acids, and xenobiotics biodegradation and metabolism, Supplementary Figure S3A. Amino acid metabolism (32.96%), carbohydrate metabolism (25.86%), nucleotide metabolism (10.17%), metabolism of cofactors and vitamins (9.42%), energy metabolism (6.33%), glycan biosynthesis and metabolism (4.55%), and lipid metabolism (4.11%) predominated all samples.

Based on KO level 3, the sequences were assigned to 183 endogenous pathways (Con_BC = 92.38%, M7_BC = 95.64%, M14_BC = 85.84%, M21_BC = 88.81% and M28_BC = 87.92%). The 12 predominant endogenous pathways (from the selected top 18) were glycolysis/gluconeogenesis [PATH:ko00010] (7.21%), citrate cycle (TCA cycle) [PATH:ko00020] (6.00%), pentose phosphate pathway [PATH:ko00030] (4.53%), pentose and glucuronate interconversions [PATH:ko00040] (4.08%), fructose and mannose metabolism [PATH:ko00051] (3.92%), galactose metabolism [PATH:ko00052] (3.42%), ascorbate and aldarate metabolism [PATH:ko00053] (2.53%), fatty acid biosynthesis [PATH:ko00061] (2.48%), fatty acid elongation [PATH:ko00062] (2.32%), fatty acid metabolism [PATH:ko00071] (2.67%), steroid biosynthesis [PATH:ko00100] (2.37%), and primary bile acid biosynthesis [PATH:ko00120] (2.29%; (Supplementary Figure S3B).

### Metabolic pathways involved in amino acids, carbohydrates, lipid and cofactors, and vitamins metabolism

**Figure 4:**
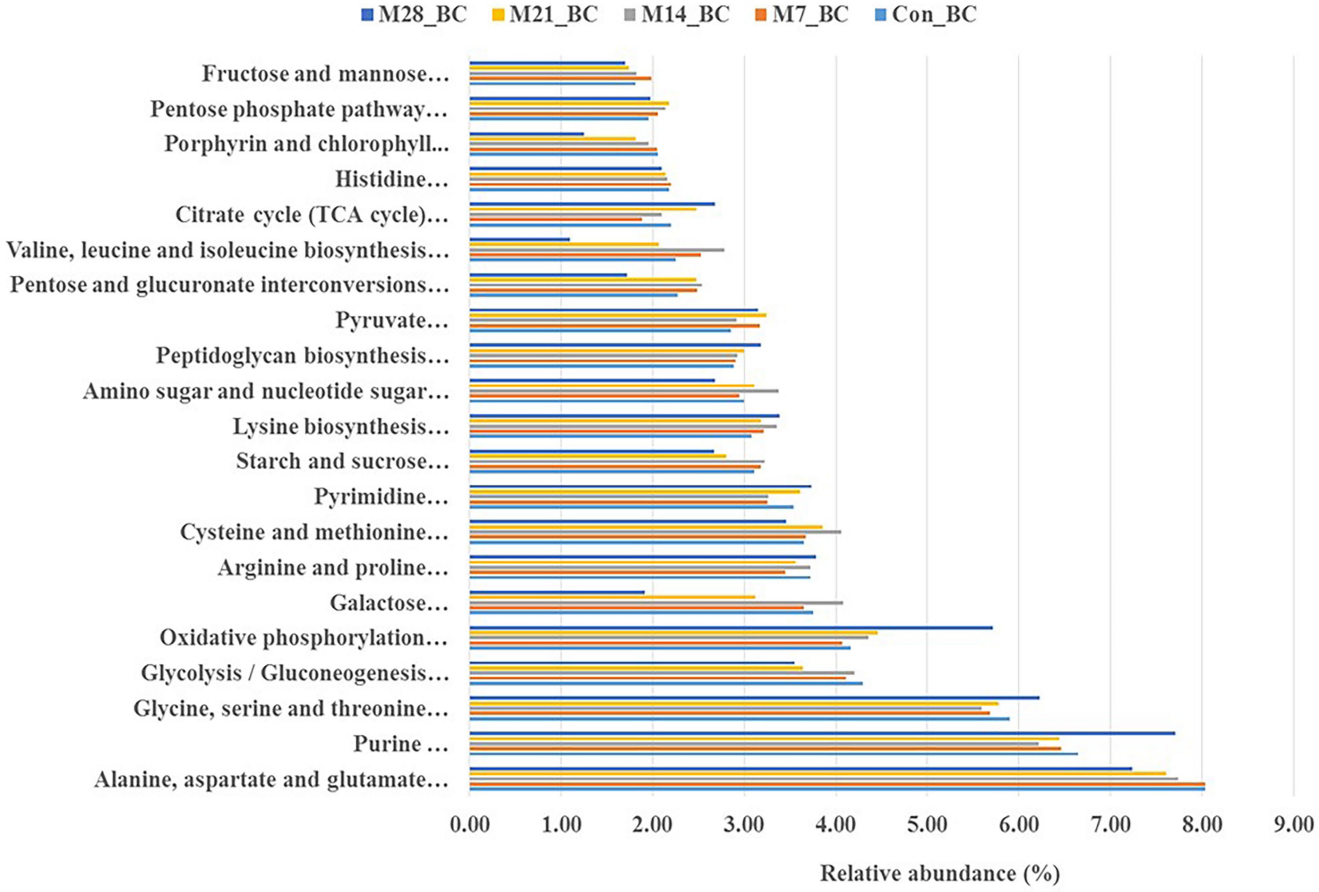
Bar plot showing relative abundance (mean > 1.02%) of the major functions (at level 1) of the microbial communities in caecum digesta samples from broiler chickens fed with diets containing different levels of marama bean meal [Treatment groups: 0% (Con_BC); 7% (M7_BC); 14% (M14_BC); 21% (M21_BC); 28% (M28_BC) inclusion levels].

The KO level 3 revealed 100 functional pathways associated with metabolism category (KO level 1). Twenty-three pathways were prominent (>1%) among all samples. Of these, twenty-one functional pathways had high relative abundance, with the mean ranging from 1.83 to 7.73%. Most of the functional potential pathways were assigned to alanine, aspartate and glutamate metabolism [PATH:ko00250], purine metabolism [PATH:ko00230], glycine, serine and threonine metabolism [PATH:ko00260], oxidative phosphorylation [PATH:ko00190], glycolysis/gluconeogenesis [PATH:ko00010] (7.73, 6.70, 5.84, 4.55, and 3.96%, respectively). As illustrated in Figure 4, the highest abundance (alanine, aspartate and glutamate metabolism [PATH: ko00250] was observed in the Con_BC (control) and M7_BC (treatment) groups, while the other three functional pathways were high in the M28_BC (treatment) group. However, based on the abundance across the treatment groups, no significant difference was observed.

#### Metabolic pathways involved in amino acids metabolism

The KO level 3 showed 13 pathways associated with amino acid metabolism (KO level 2), with four amino acid metabolic pathways being the most dominant (mean ranging from 10.95 to 23.44%) in the treatment groups. These include alanine, aspartate and glutamate metabolism [PATH:ko00250] (23.44%), glycine, serine and threonine metabolism [PATH:ko00260] (17.72%), and cysteine and methionine metabolism [PATH:ko00270] (11.34%) arginine and proline metabolism [PATH:ko00330] (10.95%). Table 5 illustrates the relative abundance of the amino acid metabolism pathways in the MBM and CON treatment groups. High relative abundance of alanine, aspartate and glutamate metabolism was observed in the M7_BC treatment group. However, there was no significant difference in the relative abundance of amino acid metabolism across all treatment groups.

**Table 5:** Relative abundance (%) of the sequences linked to amino acids metabolism of pathways (KO level_3) in the caecum digesta samples from broiler chickens fed with diets containing different levels of marama bean meal.

Amin acids metabolism pathways	Con_BC	M7_BC	M14_BC	M21_BC	M28_BC
Alanine, aspartate, and glutamate metabolism [PATH:ko00250]	23.96	24.06	23.10	23.25	22.81
Glycine, serine, and threonine metabolism [PATH:ko00260]	17.59	17.02	16.69	17.66	19.65
Proline metabolism [PATH:ko00330]	11.00	10.23	11.03	10.73	11.78
Cysteine and methionine metabolism [PATH:ko00270]	10.90	10.98	12.13	11.80	10.90
Lysine biosynthesis [PATH:ko00300]	9.18	9.63	10.03	9.73	10.67
Valine, leucine, and isoleucine biosynthesis [PATH:ko00290]	6.71	7.56	8.33	6.33	3.47
Histidine metabolism [PATH:ko00340]	6.51	6.58	6.44	6.54	6.63
Phenylalanine, tyrosine, and tryptophan biosynthesis [PATH:ko00400]	5.12	6.26	5.18	6.10	5.03
Valine, leucine, and isoleucine degradation [PATH:ko00280]	4.17	3.58	3.80	4.29	5.43
Phenylalanine metabolism [PATH:ko00360]	1.61	1.80	1.31	1.46	0.83
Tyrosine metabolism [PATH:ko00350]	1.44	1.72	1.16	1.29	1.75
Lysine degradation [PATH:ko00310]	0.97	0.31	0.45	0.33	0.21
Tryptophan metabolism [PATH:ko00380]	0.84	0.28	0.36	0.49	0.84

Treatment groups: 0% (Con_BC); 7% (M7_BC); 14% (M14_BC); 21% (M21_BC); 28% (M28_BC) inclusion levels.

#### Metabolic pathways involved in carbohydrates metabolism

Within the carbohydrate metabolism category, 15 pathways associated with carbohydrates were observed (Table 6). Five functional metabolic pathways showed high (mean ˃ 10%) relative abundance across all treatment and control groups. These include glycolysis/gluconeogenesis [PATH:ko00010], galactose metabolism [PATH:ko00052], pyruvate metabolism [PATH:ko00620], amino sugar and nucleotide sugar metabolism [PATH:ko00520], and starch and sucrose metabolism [PATH:ko00500] (with the mean 15.32, 12.63, 11.93, 11.66 and 11.59%, respectively). However, there was no significant difference in carbohydrates metabolic pathways among the treatment groups (MBM and CON).

**Table 6:** Relative abundance (%) of sequences associated with carbohydrates metabolism pathways (KO level_3) in the caecum digesta samples from broiler chickens fed with diets containing different levels of marama bean meal.

Carbohydrates metabolism pathways	Con_BC	M7_BC	M14_BC	M21_BC	M28_BC
Glycolysis/Gluconeogenesis [PATH:ko00010]	16.19	15.40	15.32	14.11	15.58
Galactose metabolism [PATH:ko00052]	14.15	13.68	14.86	12.07	8.40
Starch and sucrose metabolism [PATH:ko00500]	11.72	11.93	11.73	10.86	11.72
Amino sugar and nucleotide sugar metabolism [PATH:ko00520]	11.29	11.03	12.28	12.00	11.72
Pyruvate metabolism [PATH:ko00620]	10.77	11.89	10.64	12.56	13.83
Pentose and glucuronate interconversions [PATH:ko00040]	8.59	9.33	9.25	9.59	7.53
Citrate cycle (TCA cycle) [PATH:ko00020]	8.29	7.04	7.66	9.60	11.76
Pentose phosphate pathway [PATH:ko00030]	7.39	7.72	7.81	8.44	8.65
Fructose and mannose metabolism [PATH:ko00051]	6.85	7.46	6.66	6.75	7.47
Glyoxylate and dicarboxylate metabolism [PATH:ko00630]	2.07	2.02	1.51	1.88	1.21
Butanoate metabolism [PATH:ko00650]	1.81	1.35	1.73	1.41	1.64
Ascorbate and aldarate metabolism [PATH:ko00053]	0.49	0.61	0.25	0.33	0.21
Inositol phosphate metabolism [PATH:ko00562]	0.23	0.35	0.18	0.27	0.21
C5-Branched dibasic acid metabolism [PATH:ko00660]	0.12	0.16	0.08	0.09	0.05
Propanoate metabolism [PATH:ko00640]	0.03	0.03	0.02	0.04	0.02

Treatment groups: 0% (Con_BC); 7% (M7_BC); 14% (M14_BC); 21% (M21_BC); 28% (M28_BC) inclusion levels.

#### Metabolic pathways involved in lipid and cofactors and vitamins metabolism

**Figure 5:**
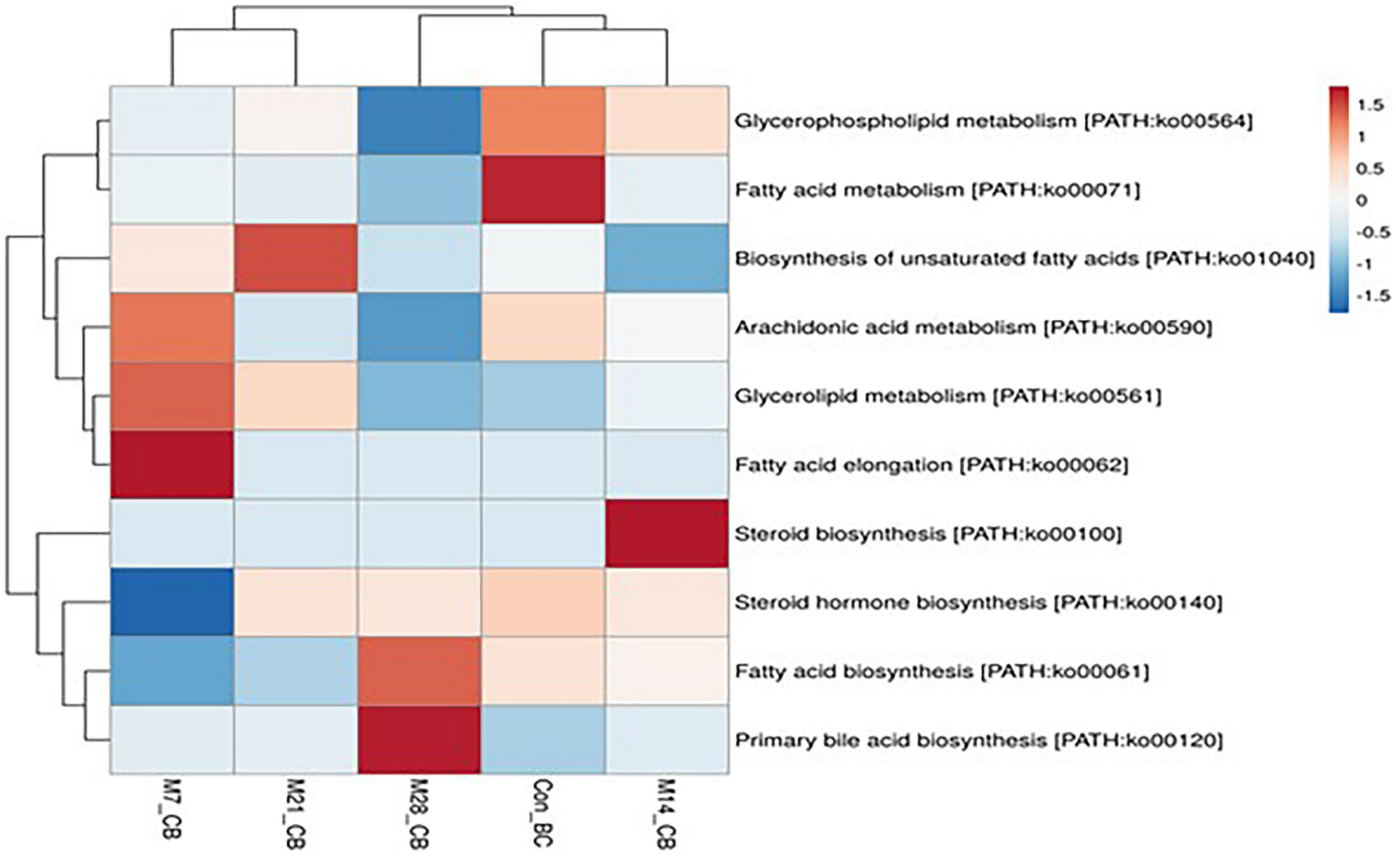
The heatmap showing relative abundance of pathways involved in lipid metabolism in the caecum digesta samples from broiler chickens fed with diets containing different levels of marama bean meal. The *X*-axis shows the treatment groups [Treatment groups: 0% (Con_BC); 7% (M7_BC); 14% (M14_BC); 21% (M21_BC); 28% (M28_BC) inclusion levels] while *Y*-axis represents metabolism pathways. The scale bar shows the colour saturation gradient dependent on the relative abundance microbial pathways. The colour intensity in each panel reflects relative abundances used for analysis (blue: low, white: medium, red: high).

**Figure 6:**
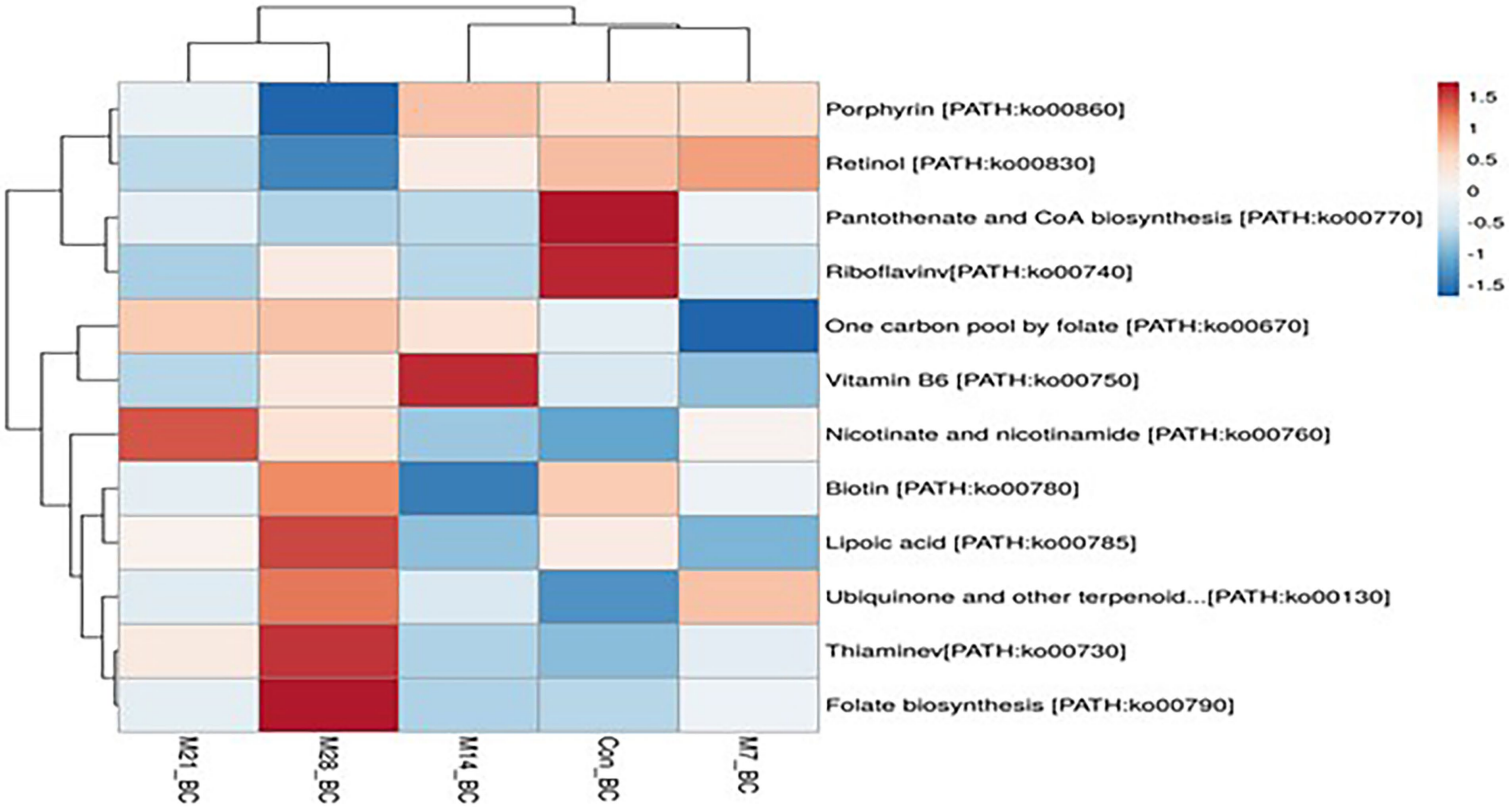
The heatmap showing relative abundance of pathways involved in Cofactors and vitamins metabolism in the caecum digesta samples from broiler chickens fed with diets containing different levels of marama bean meal. The *X*-axis shows the treatment groups [Treatment groups: 0% (Con_BC); 7% (M7_BC); 14% (M14_BC); 21% (M21_BC); 28% (M28_BC) inclusion levels] while *Y*-axis represents metabolism pathways. The scale bar shows the colour saturation gradient dependent on the relative abundance of the microbial pathways. The colour intensity in each panel reflects relative abundances used for analysis (blue: low, white: medium, red: high).

A total of 11 pathways linked to the lipid metabolism category (KO level 2) were identified (Figure 5). The most abundant (>2%) lipid metabolic pathways were fatty acid biosynthesis [PATH:ko00061], glycerophospholipid metabolism [PATH:ko00564], glycerolipid metabolism [PATH:ko00561], primary bile acid biosynthesis PATH:ko00120], and steroid hormone biosynthesis [PATH:ko00140] (with the mean being 42.72, 29.36, 19.39, 4.79, and 2.58%, respectively). However, 12 cofactors and vitamins metabolic pathways were recovered in caecum digesta samples (Figure 6). These included porphyrin [PATH:ko00860] (19.66%), nicotinate and nicotinamide [PATH:ko00760] (18.75%), one carbon pool by folate [PATH:ko00670] (11.11%), pantothenate and CoA biosynthesis [PATH:ko00770] (10.16%), thiamine [PATH:ko00730] (10.08%), riboflavin [PATH:ko00740] (7.35%), biotin [PATH:ko00780] (5.49%), vitamin B6 [PATH:ko00750] (5.32%), folate biosynthesis [PATH:ko00790] (5.22%), ubiquinone and other terpenoid-quinone biosynthesis [PATH:ko00130] (5.21%), lipoic acid [PATH:ko00785] (1.59%), and retinol [PATH:ko00830] (0.03%) were significantly enriched in caecum digesta samples.

#### Carbohydrate-degrading enzymes detected in the caecum samples

A total of 1,315 gene sequences encoded enzymes associated with 10 metabolic pathways (amino acid metabolism, biosynthesis of other secondary metabolites, carbohydrates metabolism, energy metabolism, glycan biosynthesis and metabolism, lipid metabolism, metabolism of cofactors and vitamins, metabolism of terpenoids and polyketides, nucleotide and metabolism and xenobiotics biodegradation and metabolism). Of these, 309 (23.49%) gene sequences encoded carbohydrate metabolism and the most dominant enzymes were beta-galactosidase [EC:3.2.1.23], starch phosphorylase [EC:2.4.1.1], 6-phosphofructokinase 1 [EC:2.7.1.11], and pyruvate, orthophosphate dikinase [EC:2.7.9.1] (with average; 1.02, 0.79, 0.75, and 0.71%, respectively; *P* ˃ 0.05; (Supplementary Table S3). Based on the CAZymes database, 13.27% of the carbohydrate degrading enzymes belonged to four families (glycoside hydrolases (GHs), glycosyl transferase (GTs), polysaccharide lyases (PLs), and carbohydrate esterases (CEs). The GHs were the most predominant class (10.36%), with GH1, GH5 and GH13 showing the highest abundance across all samples (Supplementary Table S4). The lowest represented categories were PLs and CEs.

## Discussion

The gut microbiota play a vital role in maintaining the intestinal health of the host and thus improve nutrient digestibility, growth performance and overall health of the birds (Yadav and Jha, 2019; Sun et al., 2021). To optimise poultry production, a thorough understanding of the structure and function of the gut microbiota, especially in chickens, is pertinent (Stanley et al., 2016). Several studies have employed high-throughput sequencing techniques to profile the gut microbiota of chickens reared on various experimental diets (Wei et al., 2013; Lee et al., 2017; Biasato et al., 2019; Qi et al., 2019; Feng et al., 2021). Despite this, no study has explored the structure and function of the caecal microbiota of broiler chickens reared on diets containing different levels of marama bean meal (MBM) as a partial alternative protein source to soybean products. The results obtained in this study revealed that bacteria domain and the phyla Bacteroides, Firmicutes, and Proteobacteria dominated the caecum digesta of both the control and the MBM treatment groups. These findings are consistent with previous studies, which assessed the microbial communities of chickens based on sex and breed (Lee et al., 2017; Du et al., 2020), chickens fed with yellow mealworm [*Tenebrio molitor* (TM)] meal or reared under different farming systems (free range and intensive) (Biasato et al., 2019; Shi et al., 2019). However, a study by Biasato et al. (2019) revealed that dietary inclusion of TM meal (at 10–15% inclusion levels) may negatively influence caecal microbiota. In this study, high abundance of the aforementioned phyla were on the birds fed with 14 and 21% MBM inclusion levels. This suggests that MBM can increase caecum microbial communities (at phyla level) in the chickens. Interestingly, these phyla are reportedly associated with growth performance in chickens (Torok et al., 2011). Given that caecum contents are composed of carbohydrates, high abundance of the Bacteroides, Firmicutes, and Proteobacteria may enhance the digestion of complex polysaccharides and, consequently, produce more energy required for growth (Torok et al., 2011; Biasato et al., 2019). Moreover, the phyla Bacteroidetes and Firmicutes are associated with short chain fatty acids (SCFA) metabolism, with Firmicutes contributing to butyrate and propionate synthesis, while Bacteroidetes contribute to the synthesis of propionate, alpha amylase, alpha-1,2-mannosidase and endo-1,4-beta-mannosidase for carbohydrates metabolism (Tan et al., 2019). Therefore, high production of essential amino acids and SCFA contribute to high energy production and reduction of pathogenic bacteria species in the caecum, while stimulating the proliferation of gut epithelial cells and villi height and, thus increase the absorptive surface area (Yadav and Jha, 2019).

*Bacteroides*, *Clostridium*, *Alistipes*, *Faecalibacterium*, *Ruminococcus*, *Eubacterium*, and *Parabacterioides* were the most abundant genera obtained in this study. These findings were consistent with the previous studies, which investigated the microbial communities in broiler chickens (Wei et al., 2013; Biasato et al., 2019; Sun et al., 2021). The abundance of *Bacteroides*, *Alistipes* and *Ruminococcus* genera were significantly higher in the MBM treatment groups (14, 21, and 28% inclusion levels) compared to the control group (basal diet). This suggests that the inclusion of MBM may have influenced the proliferation of these genera. Notably, these species are responsible for the fermentation of carbohydrates and production of butyrate in chickens (Stanley et al., 2013). Given that *Bacteroides* contributes to disease resistance in chickens (Ma et al., 2021), high number population of this genus suggests that MBM may indirectly prevents enteric diseases in chickens. Furthermore, *Ruminococcus* is capable of producing butyrate and other SCFA, such as acetic and succinic acids, through glucose metabolism and cellulose digestion, while *Alistipes* genus can produce acetic acid through the synthesis of fibrinolyisin, digestion of gelatin and fermentation of carbohydrates (Biasato et al., 2019). Therefore, these findings demonstrate that MBM does not only act as a protein source, but can also increase beneficial microbial communities and thus contribute to high production of butyrate and SCFAs, which are necessary for the chicken growth.

Based on the functional annotation, 28 functional categories were found, with most of the sequences being associated with carbohydrates. These findings are not surprising since caecum digesta is composed of carbohydrates (Sergeant et al., 2014). The other predominant functional categories found in this study were clustering-based subsystems, protein metabolism, amino acids and derivatives, DNA metabolism, miscellaneous, RNA metabolism, cofactors, vitamins, prosthetic groups, pigments, cell wall and capsule, nucleosides and nucleotides, respiration, and virulence, diseases and defence. Although the α-diversity revealed a significant difference in the functional categories at subsystem level 1 and 3, higher diversity was observed in the treatment groups compared to the control (subsystem level 1), thus indicating high diversity. On the hand, the β-diversity showed that there was no significant difference between the treatment groups and control.

The KO is the most frequently used database to study functional pathways of the microbial population. The three levels of classification (KO level 1, 2, and 3) against this database have been used for profiling the functional pathways of the microbial communities in a particular area of interest (Kumar et al., 2020). In this study, KO database (level 1) predicted six functional pathways, which include metabolism, genetic information processing, environmental information processing, cellular processes, human diseases, and organismal systems. Metabolism was the most predominant functional pathway observed in this study. This suggests that microbial communities found in the caecum contribute to the metabolism process of feed. This notion is supported by the high abundance of amino acid, carbohydrates, nucleotide, cofactors and vitamins, lipid, and energy metabolism, as well as glycan biosynthesis and metabolism found in this study. Furthermore, the glycolysis/gluconeogenesis [PATH:ko00010], citrate cycle (TCA cycle) [PATH:ko00020], pentose phosphate pathway [PATH:ko00030] were the most abundant functional pathways observed at KO (level 3). The glycolysis/gluconeogenesis and pyruvate may act as an intermediate link between carbon metabolism and amino acid metabolism (Zhang et al., 2020). In addition, KO (level 3) databases showed that 100 functional pathways were associated with metabolism (KO (level 1). Notably, the alanine, aspartate and glutamate metabolism [PATH:ko00250], and purine metabolism [PATH:ko00230] were the most commonly found metabolism pathways.

Amino acids, carbohydrates, lipid, and cofactors and vitamins play vital roles in poultry production and a clear understanding of their metabolic pathways is very pertinent in improving chicken productivity. In this study, the sequences were linked to amino acids, carbohydrates, lipid and cofactors and vitamins metabolic pathways (KO level 3). The metabolism of alanine, aspartate and glutamate [PATH:ko00250], glycine, serine and threonine [PATH:ko00260], arginine and proline [PATH:ko00330] and cysteine and methionine[PATH:ko00270] were the most predominant pathways within the amino acids metabolic pathways. In addition, within carbohydrates metabolic pathways, the metabolism for glycolysis/gluconeogenesis [PATH:ko00010], galactose [PATH:ko00052], pyruvate[PATH:ko00620], amino sugar and nucleotide sugar [PATH:ko00520] and starch and sucrose [PATH:ko00500] were the most predominant pathways. The high abundance of sequences associated with these metabolic pathways indicates high activity of degradation of complex substrates (carbohydrates) in the chicken caecum. Moreover, porphyrin [PATH:ko00860], nicotinate, and nicotinamide [PATH:ko00760]), one carbon pool by folate [PATH:ko00670] (11.11%), pantothenate and CoA biosynthesis [PATH:ko00770] and thiamine [PATH:ko00730], which were associated with lipid and cofactors and vitamins metabolic pathways, were found in all treatment groups.

The family classification of carbohydrate active enzymes and annotation of the constitutive modules offers a better understanding of the structural features of the enzymes, their intra-evolutionary relationship, and mechanic properties (Cantarel et al., 2009; Drula et al., 2022). For this reason, several studies have used the CAZymes database to classify the enzymes associated with carbohydrate metabolism (Feng et al., 2021; Yu et al., 2022). In this study, genes encoding enzymes responsible for carbohydrate metabolism were obtained. The beta-galactosidase [EC:3.2.1.23], starch phosphorylase [EC:2.4.1.1], 6-phospho fructokinase 1 [EC:2.7.1.11], and pyruvate orthophosphate dikinase [EC:2.7.9.1] enzymes were the most abundant enzymes associated with carbohydrate metabolic pathways. Based on the CAZymes database, the carbohydrates-active enzymes reported in this study belong to four different families, namely; glycoside hydrolases (GHs), glycosyl transferase (GTs), polysaccharide lyases (PLs), and carbohydrate esterases (CEs). Interestingly, most of the enzymes were ascribed to the GHs family, especially GH13, followed by GH1, GH4 and GH5. This was consistent with the previous studies, where high abundance of GH class such as GH1 and GH13 in the chicken gut using shotgun metagenomics approach was reported (Feng et al., 2021; Segura-Wang et al., 2021). The enzymes belonging to this family are responsible for the hydrolysis of glycosidic bonds found in complex carbohydrates and starch (Cantarel et al., 2009; Segura-Wang et al., 2021). This suggests that a high abundance of the enzymes from this family may increase fermentation of complex carbohydrates in the caecum, and thus produces more energy for growth in chickens. Therefore, substituting soybean with MBM may improve CAZymes and increase poultry production.

## Conclusion

To the best of our knowledge, this is the first study to report caecal microbial communities and functional pathways in broiler chickens fed with diets containing marama bean meal. The results revealed that the caeca harbor diverse microbial communities, especially at the genus level across the groups (control and treatment groups), thus indicating that dietary MB meal improves caecal microbiota. In addition, SEED subsystem analysis showed that the caecal microbial communities are associated with the degradation of complex carbohydrates and protein metabolism. The KO database predicted various metabolic pathways that are linked with amino acids, carbohydrates, lipid and cofactors and vitamin metabolism, with glycoside hydrolases (GH1, GH5, and GH13) being the most abundant carbohydrates-active enzymes. These findings suggest that dietary MB meal is an alternative protein source to soybean meal.

## Data availability statement

The raw sequence data have been deposited in the NCBI database repository, under Bioproject accession number PRJNA818149. The downstream analyses files generated from the raw data are presented in the Figshare repository, the link is: https://figshare.com/s/87bc298dd99c2ca5a1fb.

## Ethics statement

The animal study was reviewed and approved by North-West University Animal Production Research Ethics Committee. Reference Number: NWU-02007-20-A5.

## Author contributions

PM: conceptualization, investigation, methodology, data curation, formal analysis, software, validation, and visualization, writing – original draft, and writing – review editing. CM: conceptualization, project administration, resources, software, validation, and writing – review and editing. AA: formal analysis, data curation, software, validation, and visualization, writing – review and editing. All authors contributed to the article and approved the submitted version.

## Funding

The financial assistance received from the Department of Animal Science of the North-West University (South Africa) is hereby acknowledged.

## Conflict of interest

The authors declare that the research was conducted in the absence of any commercial or financial relationships that could be construed as a potential conflict of interest.

## Publisher’s note

All claims expressed in this article are solely those of the authors and do not necessarily represent those of their affiliated organizations, or those of the publisher, the editors and the reviewers. Any product that may be evaluated in this article, or claim that may be made by its manufacturer, is not guaranteed or endorsed by the publisher.

## Footnotes

^1^ https://www.mg-rast.org

^2^ https://biit.cs.ut.ee/clustvis/

^3^ http://huttenhower.sph.harvard.edu/galaxy
