# Supplementary material for: Caecal microbial communities, functional diversity, and metabolic pathways in Ross 308 broiler chickens fed with diets containing different levels of Marama (Tylosema esculentum) bean meal
Source: Front Microbiol. 2022 Oct 20;13:1009945. doi: 10.3389/fmicb.2022.1009945 (PMC9630332; doi:10.3389/fmicb.2022.1009945)
Supplement: Supplementary file 1 [file Data_Sheet_1.docx]

**Caecum Microbial Communities of Broiler Chickens Fed With In-Feed Marama Bean Diet**

Peter Kotsoana Montso^1^, Caven Mguvane Mnisi ^1,2,^, Ayansina Segun Ayangbenro^1^

^1^ Food Security and Focus area, Faculty of Natural and Agricultural Science, North-West University, Private Bag X2046, Mmabatho, 2735, South Africa

^2^ Department of Animal Science, School of Agricultural Sciences, Faculty of Natural and Agricultural Sciences, North-West University, Private Bag X2046, Mmabatho, 2735, South Africa

**Supplementary Materials**

**Table S1:** Analysis of the metagenomics sequence data from the caecum of the broiler chickens fed with in-feed Marama bean diet.

| **Analysis Statistics** | **Con_BC** | **M7_BC** | **M14_BC** | **M21_BC** | **M28_BC** |
| --- | --- | --- | --- | --- | --- |
|  | Pre-processed reads | | | | |
| Base pair Count | 438,705,958 | 959,417,806 | 307,121,028 | 419,064,879 | 817,460,557 |
| Sequences Count | 1,880,850 | 4,205,050 | 1,319,561 | 1,843,022 | 3,557,783 |
| Mean Sequence Length (bp) | 233 ± 39 | 228 ± 39 | 233 ± 39 | 227 ± 39 | 230 ± 40 |
| Mean GC content (%) | 56 ± 10 | 54 ± 10 | 56 ± 10 | 55 ± 11 | 55 ± 11 |
| Artificial Duplicate Reads: Sequence Count | 564,319 | 1,385,061 | 397,221 | 443,561 | 1,233,945 |
|  | Post-processed reads | | | | |
| Base pair Count | 301,468,322 | 630,069,388 | 210,840,093 | 311,488,825 | 527,397,719 |
| Sequences Count | 1,302,880 | 2,790,421 | 912,871 | 1,379,900 | 2,294,021 |
| Mean Sequence Length (bp) | 231 ± 40 | 226 ± 40 | 231 ± 40 | 226 ± 40 | 230 ± 40 |
| Mean GC content (%) | 56 ± 10 | 54 ± 10 | 56 ± 10 | 55 ± 11 | 55 ± 11 |
| Predicted Protein Features | 730,753 | 1,595,002 | 508,985 | 807,43 | 883,484 |
| Predicted rRNA Features | 4,076 | 8,258 | 2,896 | 4,294 | 5,109 |
| Alignment: Identified Protein Features | 440,867 | 973,301 | 322,616 | 500,823 | 490,304 |
| Alignment: Identified rRNA Features | 2,346 | 4,787 | 1,735 | 2,524 | 2,679 |

**Key:** Treatment groups: 0% (Con_BC); 7 % (M7_BC); 14 % (M14_BC); 21 % (M21_BC); 28 % (M28_BC) inclusion levels.

**Table S2:** The relative abundance of the functional categories (Subsystem level-3) of the selected top 20 of microbial communities in the caecum digesta samples from the broiler chickens

| **Functional categories** | **Con_BC (%)** | **M7_BC (%)** | **M14_BC (%)** | **M21_BC (%)** | **M28_BC (%)** |
| --- | --- | --- | --- | --- | --- |
| DNA_repair,_UvrABC_system | 1,11 | 0,90 | 0,92 | 1,05 | 1,32 |
| Ribosome_LSU_bacterial | 1,10 | 1,08 | 1,25 | 1,19 | 1,40 |
| Sugar_utilization_in_Thermotogales | 1,07 | 1,34 | 1,18 | 1,17 | 0,67 |
| De_Novo_Purine_Biosynthesis | 0,97 | 0,93 | 0,92 | 1,02 | 1,15 |
| RNA_polymerase_bacterial | 0,96 | 0,88 | 0,99 | 0,92 | 1,04 |
| DNA-replication | 0,95 | 0,92 | 0,90 | 0,94 | 1,05 |
| Maltose_and_Maltodextrin_Utilization | 0,94 | 0,89 | 1,11 | 0,73 | 0,64 |
| Peptidoglycan_Biosynthesis | 0,92 | 0,92 | 0,90 | 0,93 | 0,97 |
| Purine_conversions | 0,92 | 0,81 | 0,86 | 0,82 | 1,00 |
| Serine-glyoxylate_cycle | 0,86 | 0,82 | 0,87 | 0,92 | 1,14 |
| Phage_integration_and_excision | 0,86 | 1,03 | 0,94 | 0,90 | 1,20 |
| DNA_repair,_bacterial | 0,86 | 0,90 | 0,79 | 0,89 | 0,83 |
| Multidrug_Resistance_Efflux_Pumps | 0,82 | 0,69 | 0,88 | 0,97 | 0,94 |
| D-Galacturonate_and_D-Glucuronate_Utilization | 0,80 | 0,84 | 0,85 | 0,72 | 0,47 |
| Methionine_Biosynthesis | 0,79 | 0,81 | 0,92 | 0,95 | 0,85 |
| Glutamine,_Glutamate,… | 0,79 | 0,80 | 0,81 | 0,90 | 0,86 |
| Phosphate_metabolism | 0,76 | 0,71 | 0,73 | 0,70 | 0,72 |
| Ribosome_SSU_bacterial | 0,74 | 0,67 | 0,83 | 0,75 | 0,87 |
| CBSS-350688.3.peg.1509 | 0,71 | 0,67 | 0,71 | 0,68 | 0,70 |
| Na(+)-translocating_NADH-quinone… | 0,67 | 0,52 | 0,66 | 0,64 | 0,84 |
| Glycolysis_and_Gluconeogenesis | 0,65 | 0,71 | 0,58 | 0,65 | 0,60 |
| tRNA_modification_Bacteria | 0,63 | 0,61 | 0,59 | 0,70 | 0,73 |
| Universal_GTPases | 0,62 | 0,58 | 0,56 | 0,58 | 0,61 |

**Key:** Treatment groups: 0% (Con_BC); 7 % (M7_BC); 14 % (M14_BC); 21 % (M21_BC); 28 % (M28_BC) inclusion levels.

**Table S3:** The relative abundance of the enzymes involved in Carbohydrates metabolic pathways in the caecum of the broiler chickens fed on Marama bean diet.

| **Enzymes** | **Con_BC (%)** | **M7_BC (%)** | **M14_BC (%)** | **M21_BC (%)** | **M28_BC (%)** |
| --- | --- | --- | --- | --- | --- |
| beta-galactosidase [EC:3.2.1.23] | 1,51 | 1,04 | 1,22 | 0,87 | 0,47 |
| starch phosphorylase [EC:2.4.1.1] | 0,80 | 0,75 | 0,89 | 0,74 | 0,77 |
| 6-phosphofructokinase 1 [EC:2.7.1.11] | 0,78 | 0,81 | 0,78 | 0,73 | 0,63 |
| pyruvate,orthophosphate dikinase [EC:2.7.9.1] | 0,71 | 0,60 | 0,62 | 0,73 | 0,86 |
| 6-phospho-beta-glucosidase [EC:3.2.1.86] | 0,67 | 0,50 | 0,77 | 0,24 | 0,28 |
| 4-alpha-glucanotransferase [EC:2.4.1.25] | 0,62 | 0,65 | 0,48 | 0,53 | 0,45 |
| transketolase [EC:2.2.1.1] | 0,61 | 0,60 | 0,64 | 0,71 | 0,63 |
| UDP-N-acetylglucosamine 1-carboxyvinyltransferase [EC:2.5.1.7] | 0,61 | 0,56 | 0,52 | 0,57 | 0,57 |
| phosphomannomutase [EC:5.4.2.8] | 0,54 | 0,36 | 0,38 | 0,50 | 0,66 |
| 1,4-alpha-glucan branching enzyme [EC:2.4.1.18] | 0,50 | 0,49 | 0,48 | 0,42 | 0,45 |
| acetate kinase [EC:2.7.2.1] | 0,47 | 0,40 | 0,42 | 0,49 | 0,48 |
| UDP-glucose 4-epimerase [EC:5.1.3.2] | 0,43 | 0,42 | 0,39 | 0,42 | 0,37 |
| glucose-6-phosphate isomerase [EC:5.3.1.9] | 0,42 | 0,42 | 0,43 | 0,38 | 0,41 |
| 2-oxoglutarate ferredoxin oxidoreductase subunit alpha [EC:1.2.7.3] | 0,42 | 0,37 | 0,39 | 0,53 | 0,58 |
| alpha-galactosidase [EC:3.2.1.22] | 0,39 | 0,55 | 0,75 | 0,45 | 0,21 |
| formate C-acetyltransferase [EC:2.3.1.54] | 0,38 | 0,59 | 0,59 | 0,56 | 0,35 |
| alpha-glucosidase [EC:3.2.1.20] | 0,41 | 0,42 | 0,55 | 0,34 | 0,20 |
| phosphoglycerate kinase [EC:2.7.2.3] | 0,39 | 0,37 | 0,41 | 0,36 | 0,40 |
| glucosamine-6-phosphate deaminase [EC:3.5.99.6] | 0,34 | 0,34 | 0,39 | 0,30 | 0,41 |

**Key:** Treatment groups: 0% (Con_BC); 7 % (M7_BC); 14 % (M14_BC); 21 % (M21_BC); 28 % (M28_BC) inclusion levels.

**Table S4:** The relative abundance of carbohydrate-active enzymes (at the family level) from the caecum of the broiler chickens fed with Marama bean diet.

| **CAZymes families** | **Degradation Enzymes** | **Con_BC (%)** | **M7_BC (%)** | **M14_BC (%)** | **M21_BC (%)** | **M28_BC (%)** |
| --- | --- | --- | --- | --- | --- | --- |
| **Glycoside Hydrolases (GHs**) | | | | | | |
| **GH1** | beta-galactosidase [EC:3.2.1.23] | 0.2034 | 0.3069 | 0.2301 | 0.2705 | 0.0940 |
|  | beta-N-acetylhexosaminidase [EC:3.2.1.52] | 0.1537 | 0.1387 | 0.0875 | 0.0974 | 0.0862 |
|  | xylan 1,4-beta-xylosidase [EC:3.2.1.37] | 0.0363 | 0.0323 | 0.0183 | 0.0216 | 0.0111 |
|  | 6-phospho-beta-galactosidase [EC:3.2.1.85] | 0.0000 | 0.0023 | 0.0000 | 0.0007 | 0.0004 |
| **GH2** | alpha-N-arabinofuranosidase [EC:3.2.1.55] | 0.1146 | 0.1791 | 0.1232 | 0.1888 | 0.1200 |
| **GH4** | alpha-glucosidase [EC:3.2.1.20] | 0.4095 | 0.4162 | 0.5456 | 0.3404 | 0.2033 |
|  | 6-phospho-beta-glucosidase [EC:3.2.1.86] | 0.0070 | 0.0430 | 0.0112 | 0.0118 | 0.0099 |
|  | galacturan 1,4-alpha-galacturonidase [EC:3.2.1.67] | 0.0000 | 0.0006 | 0.0010 | 0.0020 | 0.0008 |
| **GH5** | maltose-6'-phosphate glucosidase [EC:3.2.1.122] | 0.0461 | 0.0449 | 0.0204 | 0.0248 | 0.0309 |
|  | alpha-amylase [EC:3.2.1.1] | 0.0426 | 0.0530 | 0.1242 | 0.0889 | 0.0882 |
|  | maltose phosphorylase [EC:2.4.1.8] | 0.0168 | 0.0188 | 0.0295 | 0.0418 | 0.0379 |
|  | lactase-phlorizin hydrolase [EC:3.2.1.108 3.2.1.62] | 0.0007 | 0.0006 | 0.0000 | 0.0007 | 0.0008 |
| **GH6** | endoglucanase [EC:3.2.1.4] | 0.0713 | 0.0789 | 0.0794 | 0.0608 | 0.0478 |
| **GH13** | 1,4-alpha-glucan branching enzyme [EC:2.4.1.18] | 0.5046 | 0.4896 | 0.4835 | 0.4169 | 0.4503 |
|  | trehalose-6-phosphate hydrolase [EC:3.2.1.93] | 0.1377 | 0.1691 | 0.2046 | 0.0954 | 0.0924 |
|  | alpha-amylase [EC:3.2.1.1] | 0.1279 | 0.0863 | 0.1140 | 0.1699 | 0.2219 |
|  | oligo-1,6-glucosidase [EC:3.2.1.10] | 0.1097 | 0.1614 | 0.1476 | 0.1196 | 0.0821 |
|  | [EC:2.4.1.-] | 0.0217 | 0.0223 | 0.0326 | 0.0608 | 0.0449 |
|  | sucrose phosphorylase [EC:2.4.1.7] | 0.0175 | 0.0210 | 0.0092 | 0.0196 | 0.0078 |
|  | cyclomaltodextrinase [EC:3.2.1.54] | 0.0091 | 0.0068 | 0.0163 | 0.0026 | 0.0045 |
|  | amylosucrase [EC:2.4.1.4] | 0.0035 | 0.0110 | 0.0041 | 0.0059 | 0.0025 |
| **GH15** | alpha,alpha-trehalase [EC:3.2.1.28] | 0.0000 | 0.0010 | 0.0000 | 0.0013 | 0.0000 |
| **GH16** | beta-galactosidase [EC:3.2.1.23] | 1.5095 | 1.0403 | 1.2154 | 0.8710 | 0.4681 |
| **GH27** | alpha-galactosidase [EC:3.2.1.22] | 0.3858 | 0.5530 | 0.7533 | 0.4548 | 0.2149 |
| **GH32** | fructan beta-fructosidase [EC:3.2.1.80] | 0.0007 | 0.0039 | 0.0031 | 0.0033 | 0.0017 |
| **GH39** | evolved beta-galactosidase subunit alpha [EC:3.2.1.23] | 0.0056 | 0.0055 | 0.0041 | 0.0046 | 0.0025 |
| **GH48** | cellulose 1,4-beta-cellobiosidase [EC:3.2.1.91] | 0.0007 | 0.0019 | 0.0000 | 0.0000 | 0.0000 |
| **GH55** | glucan 1,3-beta-glucosidase [EC:3.2.1.58] | 0.0007 | 0.0162 | 0.0010 | 0.0020 | 0.0000 |
| **GH57** | 4-alpha-glucanotransferase [EC:2.4.1.25] | 0.6220 | 0.6493 | 0.4774 | 0.5253 | 0.4528 |
|  | alpha-galactosidase [EC:3.2.1.22] | 0.0510 | 0.0647 | 0.0611 | 0.0562 | 0.0482 |
| **GH68** | beta-fructofuranosidase [EC:3.2.1.26] | 0.1873 | 0.2131 | 0.2871 | 0.1333 | 0.1035 |
| **GH94** | cellobiose phosphorylase [EC:2.4.1.20] | 0.0028 | 0.0039 | 0.0020 | 0.0020 | 0.0017 |
| **GlycosylTransferases (GTs)** | | | | | | |
| **GT2** | cellulose synthase (UDP-forming) [EC:2.4.1.12] | 0.0042 | 0.0126 | 0.0031 | 0.0091 | 0.0107 |
| **GT4** | [EC:2.4.1.-] | 0.0217 | 0.0223 | 0.0326 | 0.0608 | 0.0449 |
| **GT5** | starch synthase [EC:2.4.1.21] | 0.2628 | 0.3208 | 0.2993 | 0.2385 | 0.1666 |
| **GT20** | trehalose 6-phosphate synthase [EC:2.4.1.15] | 0.0000 | 0.0003 | 0.0000 | 0.0000 | 0.0000 |
| **GT35** | starch phosphorylase [EC:2.4.1.1] | 0.7995 | 0.7450 | 0.8856 | 0.7410 | 0.7740 |
| **Polysaccharide Lyases (PLs)** | | | | | | |
| **PL1** | pectate lyase [EC:4.2.2.2] | 0.0084 | 0.0110 | 0.0010 | 0.0052 | 0.0029 |
| **PL22** | oligogalacturonide lyase [EC:4.2.2.6] | 0.0091 | 0.0213 | 0.0132 | 0.0418 | 0.0829 |
| **PL5** | poly(beta-D-mannuronate) lyase [EC:4.2.2.3] | 0.0084 | 0.0029 | 0.0020 | 0.0274 | 0.0210 |
| **Carbohydrate Esterases (CEs)** | | | | | | |
| **CE4** | chitin deacetylase [EC:3.5.1.41] | 0.0000 | 0.0013 | 0.0000 | 0.0007 | 0.0008 |

**Key:** Treatment groups: 0% (Con_BC); 7 % (M7_BC); 14 % (M14_BC); 21 % (M21_BC); 28 % (M28_BC) inclusion levels.


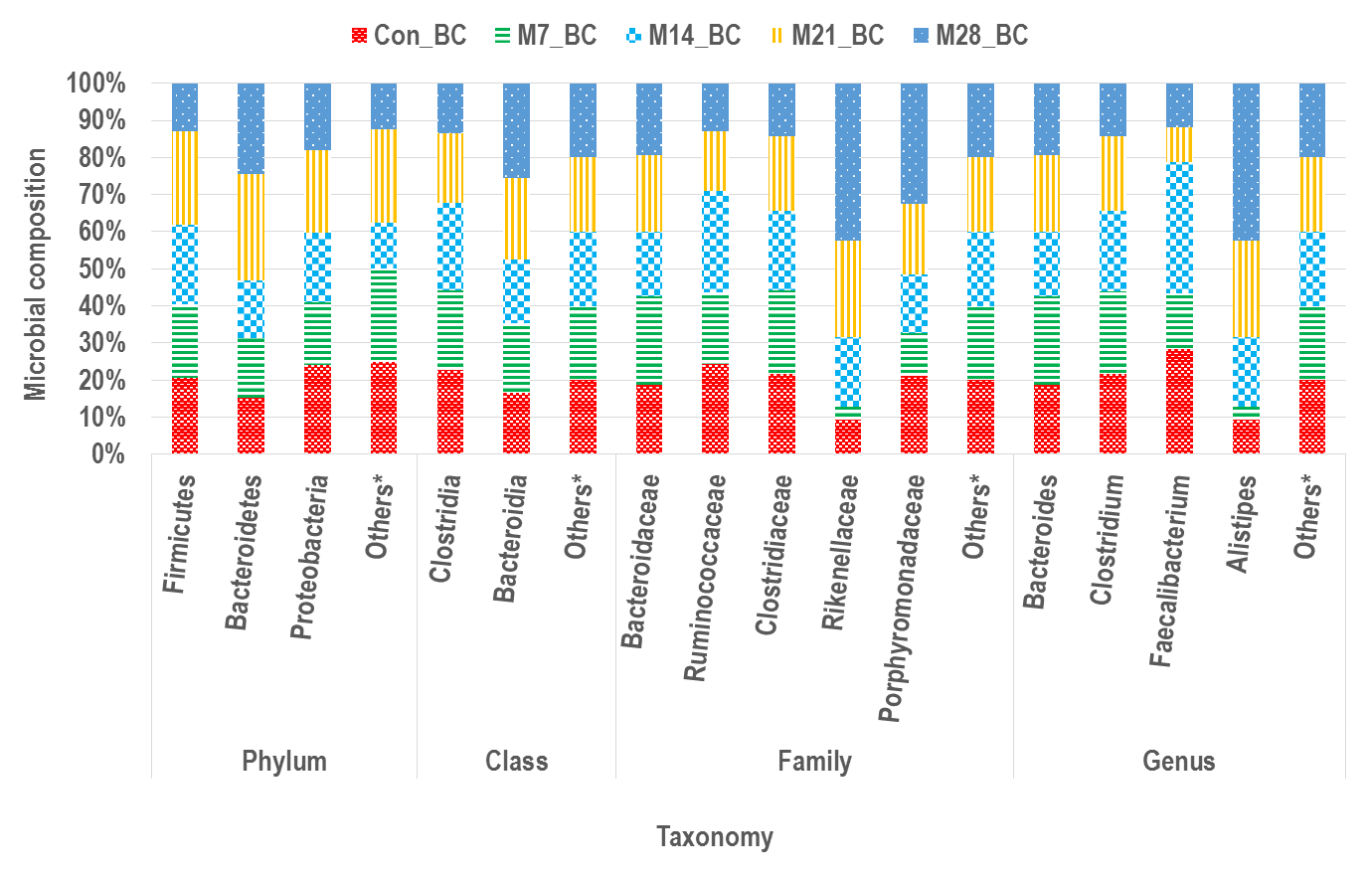


**Fig.S1.** The structure of the microbiota in the caecum digesta samples from broiler chickens fed with diets containing different levels of marama bean meal [Treatment groups: 0% (Con_BC); 7 % (M7_BC); 14 % (M14_BC); 21 % (M21_BC); 28 % (M28_BC) inclusion levels]. Asterisk (*) denotes the mean of the taxa with the percentage of each being below 4%.


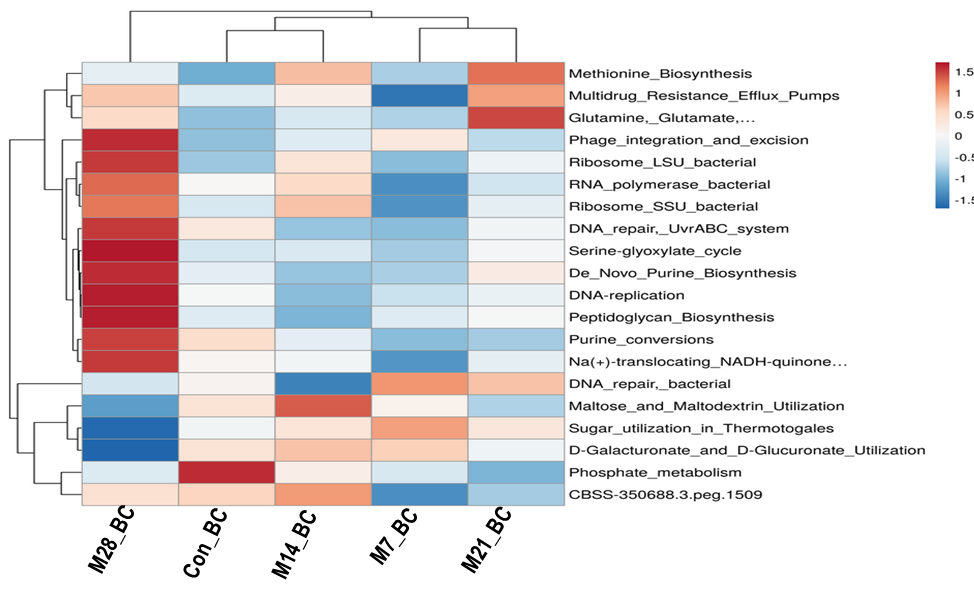


**Fig.S2.** The heatmap representation of relative abundance (mean > 1 %) of the functions at level-3 (selected top 20) of the microbial community in the caecum digesta samples from broiler chickens fed with diets containing different levels of marama bean meal [Treatment groups: 0% (Con_BC); 7 % (M7_BC); 14 % (M14_BC); 21 % (M21_BC); 28 % (M28_BC) inclusion levels]. The scale bar shows the colour saturation gradient dependent on the relative abundance of the microbial pathways. The colour intensity in each panel reflects relative abundances used for analysis (blue: low, white: medium, red: high).


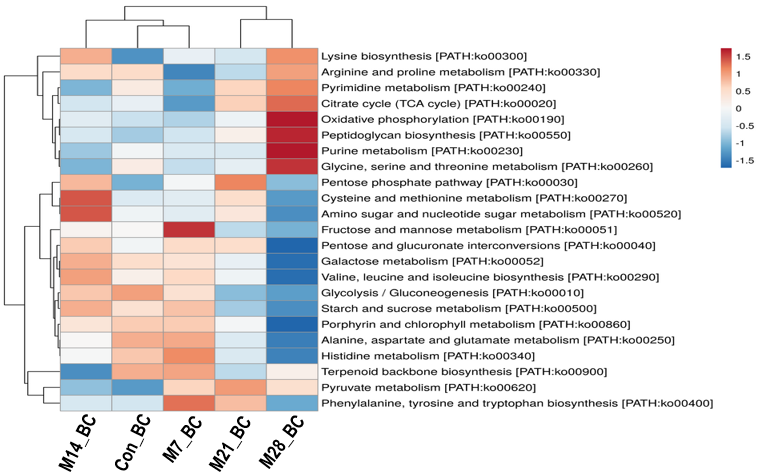


**Fig.S3a:** The heatmap showing relative abundance of the major metabolism pathways (KO level-2) of microbial communities in the caecum digesta samples from broiler chickens fed with diets containing different levels of marama bean meal [Treatment groups: 0% (Con_BC); 7 % (M7_BC); 14 % (M14_BC); 21 % (M21_BC); 28 % (M28_BC) inclusion levels]. The scale bar shows the colour saturation gradient dependent on the relative abundance of the microbial pathways**.** The colour intensity in each panel reflects relative abundances used for analysis (blue: low, white: medium, red: high).


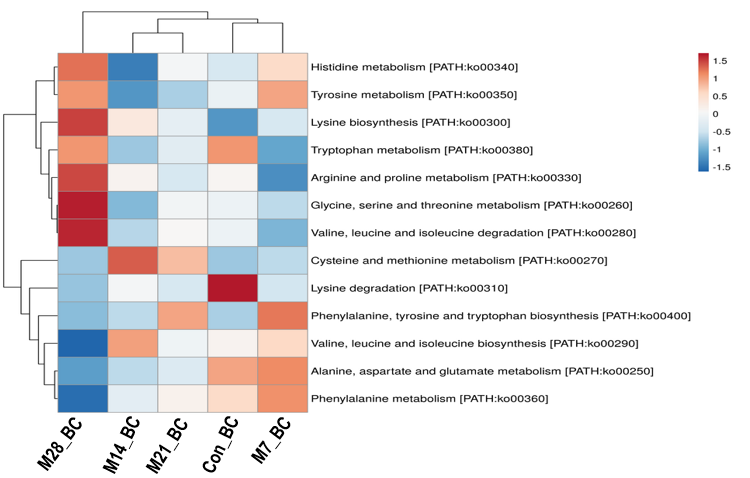


**Fig.S3b:** The heatmap showing relative abundance (> %) of the selected metabolism pathways (KO level-3) of microbial communities in the caecum digesta samples from broiler chickens fed with diets containing different levels of marama bean meal [Treatment groups: 0% (Con_BC); 7 % (M7_BC); 14 % (M14_BC); 21 % (M21_BC); 28 % (M28_BC) inclusion levels]. The scale bar shows the colour saturation gradient dependent on the relative abundance of the microbial pathways**.** The colour intensity in each panel reflects relative abundances used for analysis (blue: low, white: medium, red: high).

**
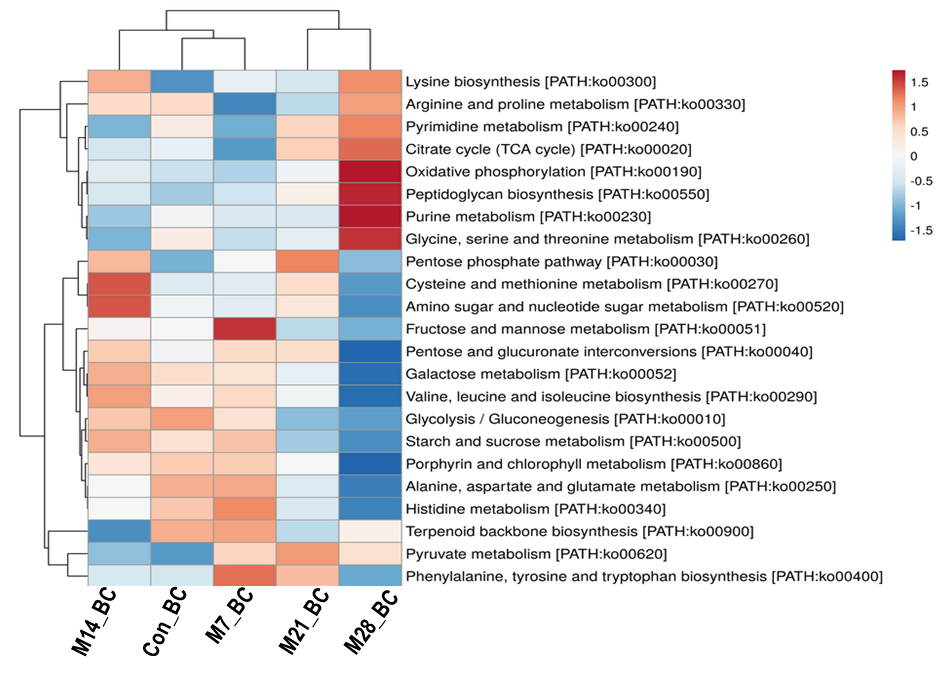
**

**Fig.S3c:** The heatmap representation of relative abundance of pathways (KO level-3) associated with carbohydrate metabolism (KO level-1 in the caecum digesta samples from broiler chickens fed with diets containing different levels of marama bean meal [Treatment groups: 0% (Con_BC); 7 % (M7_BC); 14 % (M14_BC); 21 % (M21_BC); 28 % (M28_BC) inclusion levels]. The scale bar shows the colour saturation gradient dependent on the relative abundance of the microbial pathways**.** The colour intensity in each panel reflects relative abundances used for analysis (blue: low, white: medium, red: high).

**
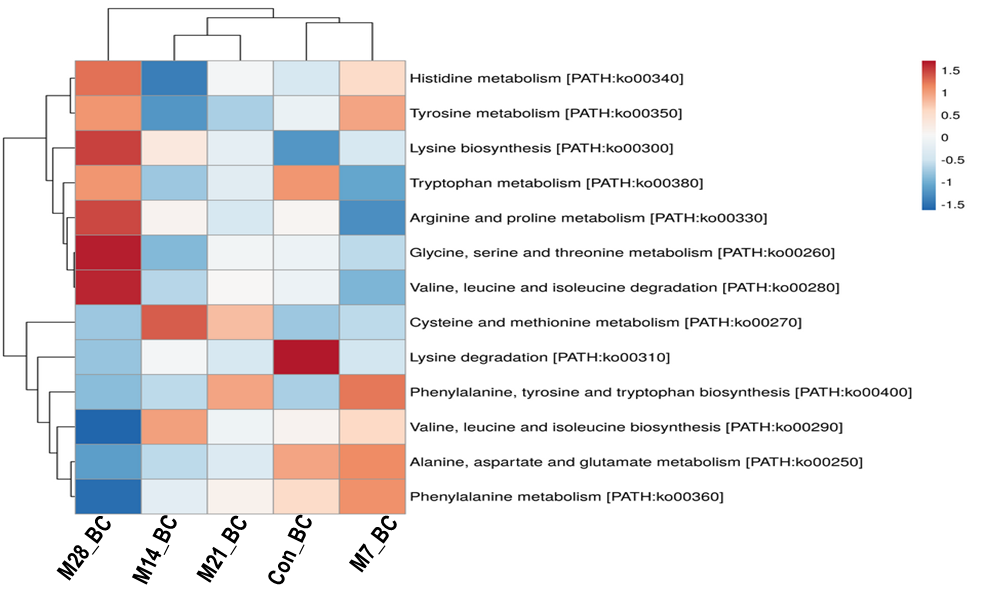
**

**Fig.S3d:** The heatmap showing pathways involved in amino acid metabolism in the caecum digesta samples from broiler chickens fed with diets containing different levels of marama bean meal [Treatment groups: 0% (Con_BC); 7 % (M7_BC); 14 % (M14_BC); 21 % (M21_BC); 28 % (M28_BC) inclusion levels]. The scale bar shows the colour saturation gradient dependent on the relative abundance of the microbial pathways**.** The colour intensity in each panel reflects relative abundances used for analysis (blue: low, white: medium, red: high).

**
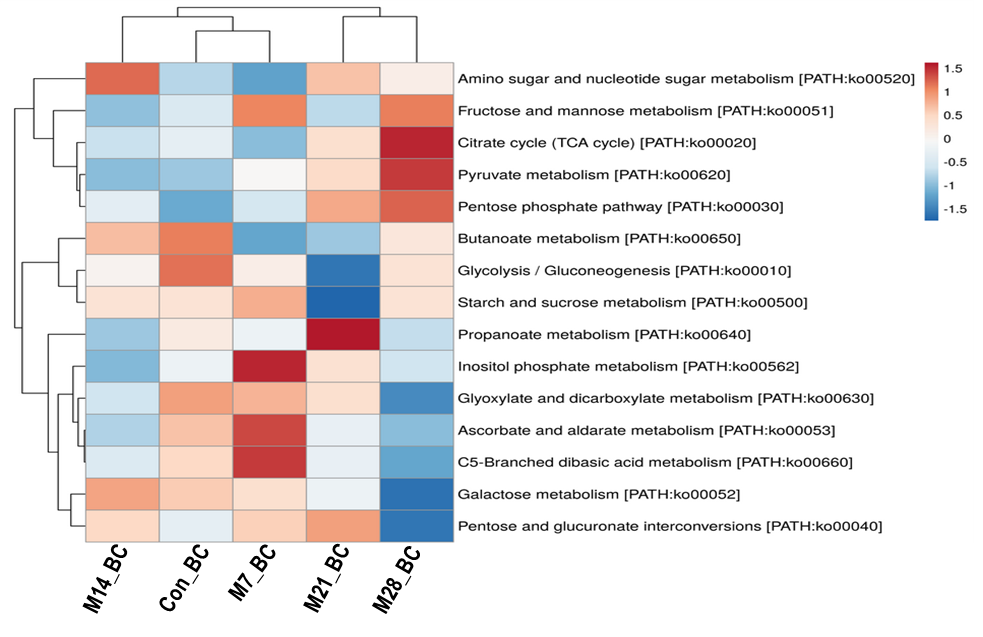
**

**Fig.S3e:** The heatmap depicting relative abundance of pathways involved in carbohydrates metabolism in the caecum digesta samples from broiler chickens fed with diets containing different levels of marama bean meal [Treatment groups: 0% (Con_BC); 7 % (M7_BC); 14 % (M14_BC); 21 % (M21_BC); 28 % (M28_BC) inclusion levels]. The scale bar shows the colour saturation gradient dependent on the relative abundance of the microbial pathways**.** The colour intensity in each panel reflects relative abundances used for analysis (blue: low, white: medium, red: high).

**
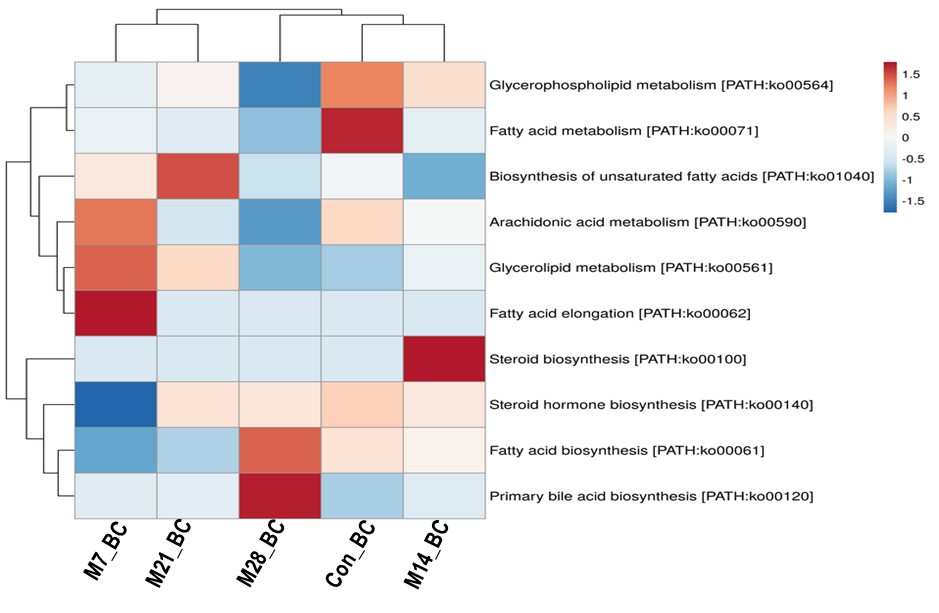
**

**Fig.S3f:** The heatmap showing relative abundance of pathways involved in lipid metabolism in the caecum digesta samples from broiler chickens fed with diets containing different levels of marama bean meal [Treatment groups: 0% (Con_BC); 7 % (M7_BC); 14 % (M14_BC); 21 % (M21_BC); 28 % (M28_BC) inclusion levels]. The scale bar shows the colour saturation gradient dependent on the relative abundance microbial pathways**.** The colour intensity in each panel reflects relative abundances used for analysis (blue: low, white: medium, red: high).

**
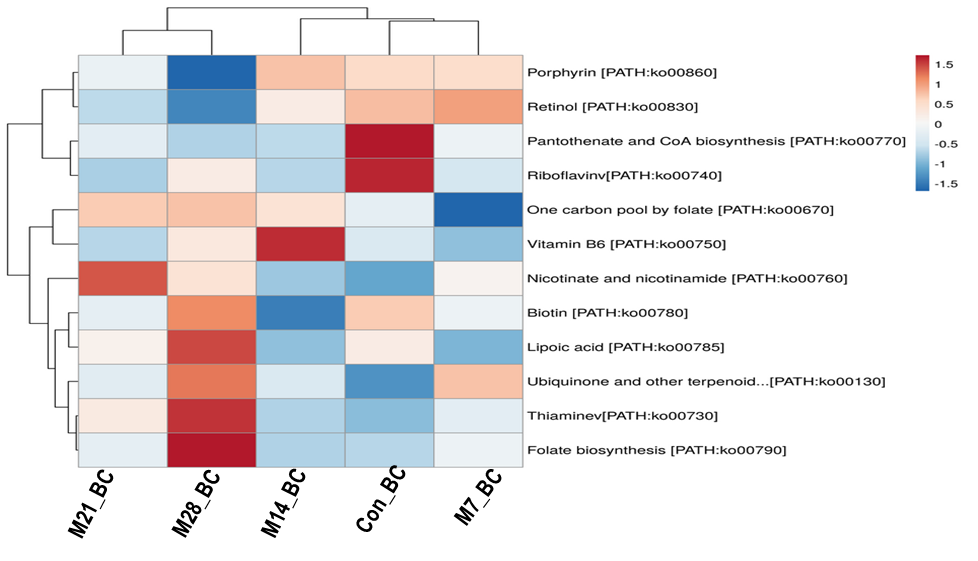
**

**Fig.S3g:** The heatmap showing relative abundance of pathways involved in Cofactors and vitamins metabolism in the caecum digesta samples from broiler chickens fed with diets containing different levels of marama bean meal [Treatment groups: 0% (Con_BC); 7 % (M7_BC); 14 % (M14_BC); 21 % (M21_BC); 28 % (M28_BC) inclusion levels]. The scale bar shows the colour saturation gradient dependent on the relative abundance of the microbial pathways**.** The colour intensity in each panel reflects relative abundances used for analysis (blue: low, white: medium, red: high).
